# Urbanization is a main driver for the larval ecology of *Aedes* mosquitoes in arbovirus-endemic settings in south-eastern Côte d'Ivoire

**DOI:** 10.1371/journal.pntd.0005751

**Published:** 2017-07-13

**Authors:** Julien B. Z. Zahouli, Benjamin G. Koudou, Pie Müller, David Malone, Yao Tano, Jürg Utzinger

**Affiliations:** 1 Swiss Tropical and Public Health Institute, Basel, Switzerland; 2 University of Basel, Basel, Switzerland; 3 Centre Suisse de Recherches Scientifiques en Côte d’Ivoire, Abidjan, Côte d’Ivoire; 4 Unité de Formation et de Recherche Biosciences, Université Félix Houphouët-Boigny, Abidjan, Côte d’Ivoire; 5 Centre for Neglected Tropical Diseases, Liverpool School of Tropical Medicine, Liverpool, United Kingdom; 6 Université Nangui-Abrogoua, Abidjan, Côte d’Ivoire; 7 Innovative Vector Control Consortium, Liverpool School of Tropical Medicine, Liverpool, United Kingdom; National Yang-Ming University, TAIWAN

## Abstract

**Background:**

Failure in detecting naturally occurring breeding sites of *Aedes* mosquitoes can bias the conclusions drawn from field studies, and hence, negatively affect intervention outcomes. We characterized the habitats of immature *Aedes* mosquitoes and explored species dynamics along a rural-to-urban gradient in a West Africa setting where yellow fever and dengue co-exist.

**Methodology:**

Between January 2013 and October 2014, we collected immature *Aedes* mosquitoes in water containers in rural, suburban, and urban areas of south-eastern Côte d’Ivoire, using standardized sampling procedures. Immature mosquitoes were reared in the laboratory and adult specimens identified at species level.

**Principal findings:**

We collected 6,159, 14,347, and 22,974 *Aedes* mosquitoes belonging to 17, 8, and 3 different species in rural, suburban, and urban environments, respectively. *Ae*. *aegypti* was the predominant species throughout, with a particularly high abundance in urban areas (99.374%). Eleven *Aedes* larval species not previously sampled in similar settings of Côte d’Ivoire were identified: *Ae*. *albopictus*, *Ae*. *angustus*, *Ae*. *apicoargenteus*, *Ae*. *argenteopunctatus*, *Ae*. *haworthi*, *Ae*. *lilii*, *Ae*. *longipalpis*, *Ae*. *opok*, *Ae*. *palpalis*, *Ae*. *stokesi*, and *Ae*. *unilineatus*. *Aedes* breeding site positivity was associated with study area, container type, shade, detritus, water turbidity, geographic location, season, and the presence of predators. We found proportionally more positive breeding sites in urban (2,136/3,374, 63.3%), compared to suburban (1,428/3,069, 46.5%) and rural areas (738/2,423, 30.5%). In the urban setting, the predominant breeding sites were industrial containers (e.g., tires and discarded containers). In suburban areas, containers made of traditional materials (e.g., clay pots) were most frequently encountered. In rural areas, natural containers (e.g., tree holes and bamboos) were common and represented 22.1% (163/738) of all *Aedes*-positive containers, hosting 18.7% of the *Aedes* fauna. The predatory mosquito species *Culex tigripes* was commonly sampled, while *Toxorhynchites* and *Eretmapodites* were mostly collected in rural areas.

**Conclusions/significance:**

In Côte d’Ivoire, urbanization is associated with high abundance of *Aedes* larvae and a predominance of artificial containers as breeding sites, mostly colonized by *Ae*. *aegypti* in urban areas. Natural containers are still common in rural areas harboring several *Aedes* species and, therefore, limiting the impact of systematic removal of discarded containers on the control of arbovirus diseases.

## Author summary

Outbreaks of yellow fever and dengue caused by *Aedes* mosquitoes have been repeatedly reported in rural and urban areas in humid tropical Africa, including Côte d’Ivoire. Although controlling immature stages of *Aedes* mosquitoes in their aquatic habitats before they become adult vectors remains the best method to fight arboviral diseases, failure to identify the larval habitats can compromise intervention success. We studied the larval ecology of *Aedes* mosquitoes in different settings (rural, suburban, and urban) in Côte d’Ivoire. We found that the degree of urbanization was significantly associated with *Aedes* breeding sites. Compared with rural areas, urban and suburban areas were characterized by high numbers of *Aedes* mosquito breeding sites; mostly artificial containers (e.g., tires and discarded containers) that were inhabited by the larvae of *Ae*. *aegypti*. In rural areas, natural containers (e.g., tree holes and bamboos) harbored several other *Aedes* species not found elsewhere. Our results suggest that removal of discarded containers–a common practice in arbovirus control programs–in urban areas does not suffice for controlling arboviral diseases because urban areas remain exposed to (re)infestation due to natural containers that host several *Aedes* species in rural areas. Additional vector control strategies are required.

## Introduction

Several *Aedes* species act as vectors of arboviral diseases, such as yellow fever, dengue, chikungunya, Rift Valley fever, and Zika virus infections that are of considerable public health relevance [1]. The transmission patterns of these arboviruses and their geographic expansion are expected to change due to environmental transformation, including urbanization [2, 3]. Besides yellow fever, other arboviruses are likely underestimated and underreported in Africa because of low awareness by health care providers, other prevalent non-malarial febrile illnesses, lack of diagnostic tests, and absence of systematic surveillance [4]. Nevertheless, yellow fever, dengue (DENV1-4), chikungunya, and Zika viruses are currently circulating in West Africa through the sylvatic, rural, and epidemic cycles maintained by wild and urban vectors [5, 6]. Côte d’Ivoire has been repeatedly facing yellow fever and dengue outbreaks involving several vectors such as *Aedes africanus*, *Ae*. *furcifer*, *Ae*. *luteocephalus*, *Ae*. *opok*, and *Ae*. *vittatus* in rural, and *Ae*. *aegypti* in urban areas [7, 8]. These outbreaks have often occurred in foci characterized by high rate of urbanization due to economic development supported by palm oil and rubber farming, trade, and traffic [7].

Arboviral disease transmission is influenced by community-level effects of container-dwelling *Aedes* mosquito larvae by regulating the production and fitness of adult vectors [9]. *Aedes* mosquito larvae are highly sensitive to environmental changes, including urbanization [10]. Some *Aedes* species (e.g., *Ae*. *aegypti*) inhabit a wide variety of containers ranging from natural containers (e.g., tree holes) to artificial containers (e.g., tires, discarded items, and other water containers) due to their ecologic plasticity [11], while others are restricted to specific breeding sites because of the higher sensibility of their offspring to environmental changes [12]. The ecologic plasticity allows *Ae*. *aegypti* and *Ae*. *albopictus* to spread worldwide by sea, air, and land transportation networks, and to adapt to new and changing environments [10].

The choice of breeding sites is governed by competition and predation among immature stages of *Aedes* and other mosquitoes that co-exist in the same breeding site [11, 12]. For example, intra- and interspecific competition between *Ae*. *aegypti* and *Ae*. *albopictus* [13] and among several *Aedes* species [12] has been reported. Moreover, mosquito species such as *Toxorhynchites* spp., *Eretmapodites* spp., and *Culex tigripes* predate on the larvae of *Aedes* [12, 13]. The biotic factors may also interact with abiotic factors, such as the climate [13]. As larvae directly depend on water, precipitation is the most important physical factor. The complex patterns of flooding and drying of larval breeding sites govern arboviral transmission [14].

In Côte d’Ivoire, yellow fever has been a key factor that forced the transfer of the colonial capital from Grand-Bassam to Bingerville near Abidjan in 1899 [15]. However, more than a century later, yellow fever and dengue outbreaks still remain an unresolved public health issue [7, 8, 15]. During arbovirus epidemics, vector controls are mostly based on the systematic removal of artificial *Aedes* breeding sites in urban areas.

The most effective vector control strategy is the control of immature stages in their aquatic habitats [12]. Hence, effective larval control requires a deep understanding of larval ecology. Our study aimed to characterize the dynamics of *Aedes* larval breeding sites, species composition, and biological associations in terms of geographic and seasonal variations along a rural-to-urban gradient in south-eastern Côte d’Ivoire. As *Aedes* mosquito larvae are highly sensitive to environmental changes [10], we hypothesized that larval breeding sites differ in species composition between urban and rural areas.

## Methods

### Ethics statement

The study protocol received approval from the local health and other administrative authorities. All entomologic surveys and sample collections carried out on private lands or private residential areas were done with the permission and written informed consent of the residents. This study did not involve endangered or protected species.

### Study area

**Fig 1:**
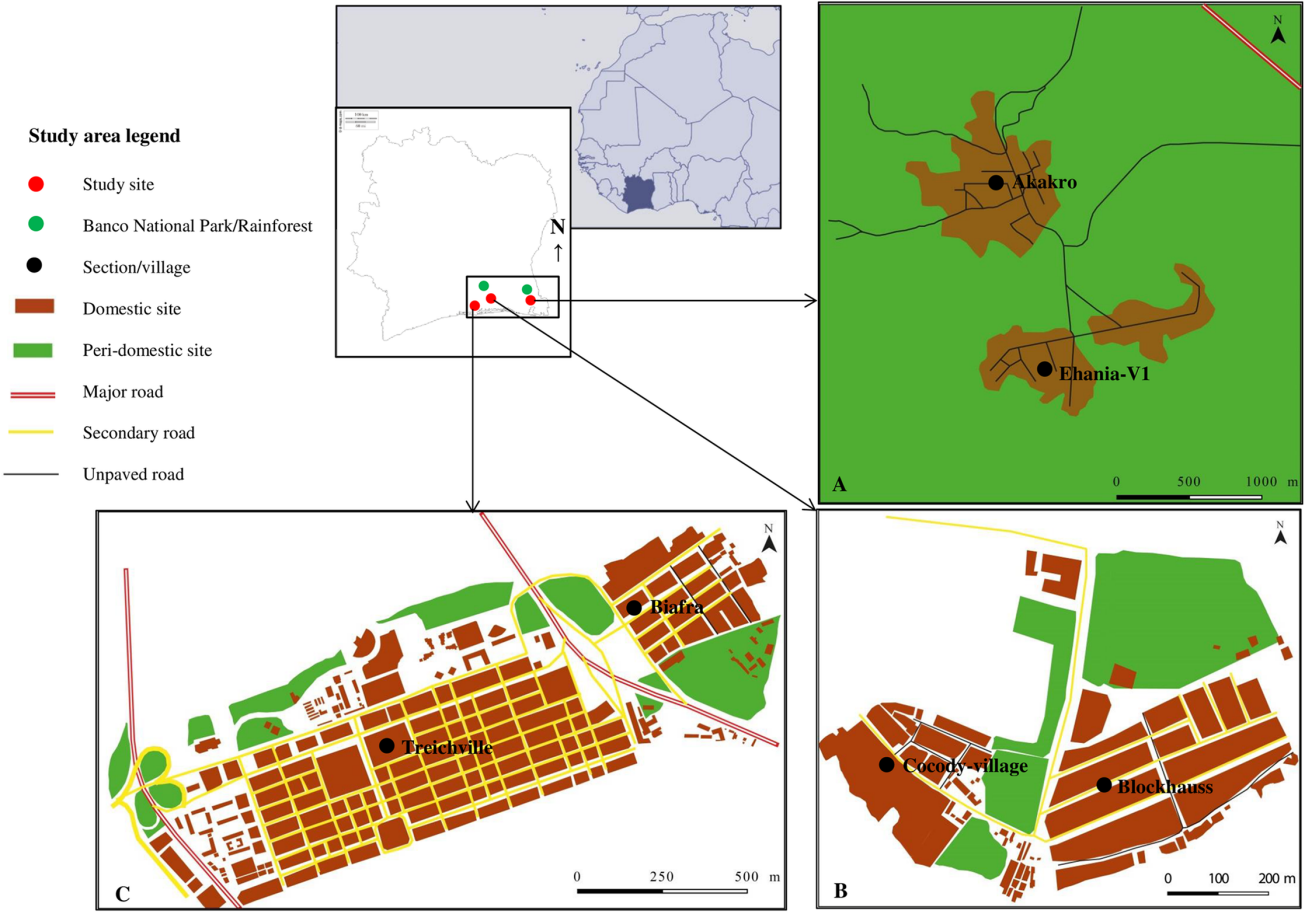
Location of the study areas in south-eastern Côte d’Ivoire. The larval breeding sites of *Aedes* mosquitoes were monitored in three areas: Ehania-V1 (A), Blockhauss (B), and Treichville (C), representing rural, suburban, and urban settings, respectively. The study site of Ehania-V1 includes the villages of Ehania-V1 and Akakro and represents the rural area without major and secondary paved roads. The site is in close proximity to a primary rainforest. The study site of Blockhauss covers the villages of Blockhauss and Petit-Cocody and represents the suburban area with only secondary paved roads. It is about 5 km away from the rainforest of the Banco National Park. The study site of Treichville comprises the sections of Jacques-Aka and Biafra and is an urban area with numerous major and secondary paved roads. It is located in the center of Abidjan and is separated from Blockhauss by the Ebrié lagoon.

The study was conducted in three areas located within a traditional arbovirus focus in south-eastern Côte d’Ivoire: Ehania-V1 (geographic coordinates 5° 18’ N latitude, 3° 4’ W longitude), Blockhauss (5° 19 N, 4° 0’ W), and Treichville (5° 18 N, 4° 0’ W), representing an increasing urbanization gradient (Fig 1). The degree of urbanization is characterized by land use, vegetation coverage, human population density, state of roads, and public services, as described in Zahouli et al. [16].

Natural and artificial containers such as tree holes, bamboo, fruit husks, tires, discarded items, and water storage receptacles that may serve as potential breeding sites for *Aedes* mosquitoes vary according to human habitation and activities. The rural area is surrounded by farms of palm oil trees (*Eleasis guineensis*) covering 11,444 ha and a preserved rainforest of 100 ha, while the suburban area is located about 2 km away from the Banco National Park with over 3,750 ha of rainforest. The rainforest is inhabited by a diverse fauna (e.g., primates and birds) that serve as hosts for *Aedes* mosquitoes.

The climate is characterized by high temperature and precipitation with two rainy seasons. The seasons are distinguished by rainfall rather than temperature. The main rainy season extends from May to July, while the shorter rainy season occurs from October to November, with distinct dry seasons in between. The average annual precipitation ranges from 1,200 to 2,400 mm. The annual average temperature and relative humidity are around 26.5°C and 80–90%, respectively.

### Study design

*Aedes* larval breeding sites were sampled quarterly in domestic (space inhabited by humans) and peri-domestic (surrounding vegetated environment within a 600 m radius from the domestic areas) sites in rural, suburban, and urban areas from January 2013 to October 2014. While water-holding containers, tree holes, and bamboo were repeatedly sampled, other potential breeding sites were sampled for the presence of immature stages of *Aedes* mosquitoes. All accessible properties were surveyed simultaneously in the three settings. Some properties could not be sampled because the residents refused to provide access or because there were physical barriers of access.

### Characterization of *Aedes* breeding sites

Potential larval breeding sites of *Aedes* mosquitoes were sampled in all three study sites by teams consisting of four trained mosquito collectors in each study area. Each mosquito collector team was composed of the same persons during all surveys. The number of experienced mosquito collectors was constant on any one day in each study area, whereas the teams made rotations from one study area to another in order to ensure similar sampling efforts and efficiency in the three study areas, and minimize potential biases. The collectors worked from 08:00 to 16:00 hours, and spent proportionally equal time periods searching for potential mosquito breeding sites in the study areas.

Readily visible and accessible containers in the selected households and surrounding premises were examined for the presence of water and mosquito larvae. In a preliminary survey, existing larval breeding sites, such as natural and artificial cavities or containers with a potential to contain water were kept in an inventory and assigned a unique label. Based on this preliminary survey, potential breeding sites were classified into two categories, three sub-categories, and 16 types, depending on their location, origin, material, and container type (Table 1 and S1 Fig). The breeding sites were assessed for abiotic and biotic characteristics, including geographic location (domestic and peri-domestic sites), color, exposure to sunlight (full shade, no exposure to sunlight; partial shade, partial exposure to sunlight; no shade, permanent exposure to sunlight), turbidity (transparent/clear, colored, opaque), substrate type (no substrate, foliage, moss, soil), surface of water, depth, presence of mosquito larvae, and predators (larvae of *Cx*. *tigripes*, *Eretmapodites* spp., and *Toxorhynchites* spp. mosquitoes, toad tadpoles, and arachnids).

**Table 1:** Classification of *Aedes* mosquito breeding sites sampled in south-eastern Côte d’Ivoire from January 2013 to October 2014.

**N°**	Breeding site	Definition
**I**	**Natural**^**a**^^**,**^^**b**^	**Containers created without or by indirect intervention of humans**
A	Rock hole^c^	Irregular and shallow shapes of massive stone of different sizes well exposed to sunlight
B	Animal detritus^c^	Debris of animal such as snail shells (carapaces of *Achatina* spp.) and animal bones
C	Tree hole^c^	Rot and pan holes of different shapes and volume located from 0 to 2 m above the ground level
D	Bamboo^c^	Cut of fixed masses of bamboos (*Bambusae*) and bars of bamboos used as fences
E	Leaf^c^	Sheathing leaf axils from plants such as bromeliads (*Ananas* spp.), bananas (*Musa* spp.) and taros (*Colocasia* spp.), and fallen sheets on the floor
F	Fruit husk^c^	Skins of coconuts (*Cocos* spp.) and flowers of bananas (*Musa* spp.)
II	**Artificial**^**a**^	**Containers created by direct intervention of humans**
**II-1**	**Traditional**^**b**^	**Handcrafted items**
G	Clay pot^c^	Ceramic containers made of clay
H	Wood-container^c^	Containers fabricated of woods such as mortars, calabashes, boats and statutes
I	Metallic pot^c^	Containers made of metals such as cooking pots
**II-2**	**Industrial**^**b**^	**Containers manufactured by factories**
J	Tarp^c^	Plastic sheets left at the ground or covering house roofs holding puddles (temporary small water collections) formed after rainfall
K	Discarded container^c^	Human waste such as broken plastic bottles, bowls, metal boxes, used cans, vases, coolers, refrigerators, shoes, and toys
L	Tire^c^	Bicycle, vehicle, and machine wheels left outdoors
M	Vehicle tank^c^	Reservoirs of abandoned cars and machines
N	Vehicle carcasses^c^	Plastic and metallic debris of abandoned cars and machines
O	Building tool^c^	Materials used to build and improve the houses such as air conditioner, bricks, metal sheets, toilets and flower pots
P	Water storage container^c^	Plastic and metallic receptacles used to store potable water

^a,b,c^: The inspected containers were grouped into the two categories^a^, three sub-categories^b^, and 16 types^c^, as defined above. The container type often reflects the name of the container.

### Mosquito sampling

Larvae and pupae of *Aedes* mosquitoes were sampled using the World Health Organization (WHO) standard equipment adapted to the aperture and the depth of larval habitats. A flexible collection tube connected to a manual suction pump was used to sample water from bromeliads and bamboo holes. Scoops of 350 ml capacity were used to collect immature mosquitoes from larger breeding sites (e.g., tree holes, discarded containers, tires, and puddles). The collected *Aedes* mosquito were counted using a pipette and classified as young larvae (1–2 instar), old larvae (3–4 instar), and pupae. Non-*Aedes* mosquito larvae such as *Anopheles* spp., *Coquelitidia* spp., *Culex* spp., *Eretmapodites* spp., *Filcabia* spp., *Toxorhynchites* spp., and *Uranotenia* spp. were also recorded. The predacious larvae of mosquitoes, such as *Cx*. *tigripes*, *Eretmapodites* spp., and *Toxorhynchites* spp. were removed from the samples to avoid predation on the other species and preserved separately. All mosquito samples were stored separately in plastic boxes and transported in a coolbox to a field laboratory.

### Laboratory procedures

In the laboratory, mosquito larvae were reared until they reached the adult stage. In order to minimize mortality, a maximum of 20 larvae were placed in 200 ml plastic cups, filled with 150 ml distilled water and covered with netting. Larvae of *Aedes* and other mosquitoes were fed each morning between 07:00 and 08:00 hours with Tetramin Baby Fish Food. Predacious larvae of *Toxorhynchites* spp. and *Cx*. *tigripes* were fed with larvae from colonies sampled from the study areas. Emerging adult mosquitoes were identified to species level using a morphological key [17]. As larval mortalities were low, the proportion of mosquito species was estimated on the basis of emerging adults. Adult specimens were stored by species and recorded in an entomology collection database.

### Statistical analysis

The frequency of *Aedes*-positive breeding sites (FP) was calculated as the percentage of water holding containers with at least one larva or pupa (numerator) among the wet containers (denominator). The proportion of *Aedes*-positive breeding site types among the *Aedes*-positive breeding sites (PP) was expressed as the percentage of each *Aedes*-positive container type (numerator) among the total *Aedes*-positive containers (denominator) in each study area. To test whether there was a difference in the number of positive breeding sites and the number of available wet containers in each category, we used Fisher’s exact test and χ^2^, as appropriate, to test for differences in the frequency of *Aedes*-positive breeding sites across the three study areas, between the domestic and peri-domestic sites, and between dry and rainy seasons.

*Aedes* species proportions were calculated as the percentage of specimens belonging to the genus *Aedes* for each study area and then compared between breeding sites as above. Larval abundances of *Aedes* mosquitoes were standardized as the mean numbers of larvae per liter of water, expressed as the geometric mean, known as Williams’ mean (i.e., log[number of mosquito larvae + 1]) [18], and compared using the Kruskal-Wallis test, followed by Mann-Whitney. The Mann-Whitney U test was also performed to compare pairs of study areas when the Kruskal-Wallis H test showed a significant difference or only two habitats. *Aedes* species richness was defined as the number of collected species in each study area and compared using a one-way analysis of variance (ANOVA), followed by the Tukey post-hoc test for post-hoc pairwise comparisons [19]. *Aedes* species diversity and dominance were estimated using the Shannon-Weaver index [20] and Simpson index [21] and analyzed using a Kruskal-Wallis test. Kruskal-Wallis test was performed because a test for normality showed a significant difference in the variances after log-transforming the data. A significance level of 5% was set for statistical testing. All statistical analyses were conducted using Stata version 14.0 (Stata Corporation; College Station, TX, United States of America).

## Results

### Mosquito species composition

Table 2 shows the species composition of adult mosquitoes that emerged from the larvae and pupae sampled from the breeding sites along the rural-to-urban gradient in south-eastern Côte d’Ivoire and reared after transfer to the laboratory. In total, 7,661, 16,931, and 26,968 adult mosquitoes emerged from the collected larvae in rural, suburban, and urban areas, respectively. The rural setting had the highest mosquito species diversity (eight genera and 37 species), followed by the suburban setting (four genera and 14 species), and the urban setting (three genera and nine species). The genus *Aedes* predominated throughout, with proportions of 80.40% (n = 7,661) in rural, 84.75% (n = 16,931) in suburban, and 85.19% (n = 26,968) in urban settings. The rural setting had the largest number of *Aedes* species (17 species), followed by the suburban (eight species) and urban settings (three species).

**Table 2:** Species composition of emerged adult mosquitoes collected as larvae in the rural, suburban and urban areas in arbovirus-endemic areas in south-eastern Côte d’Ivoire from January 2013 to October 2014.

Genus	Species	Rural				Suburban				Urban			
		F	M	T	%	F	M	T	%	F	M	T	%
***Aedes***	*Ae*. *aegypti*	2331	2296	4627	60.40	6651	6827	13478	79.61	11526	11303	22829	84.65
	*Ae*. *africanus*	69	74	143	1.87	0	0	0	0.00	0	0	0	0.00
	*Ae*. *albopictus*	0	0	0	0.00	0	0	0	0.00	2	0	2	0.01
	*Ae*. *angustus*	14	19	33	0.43	23	20	43	0.25	0	0	0	0.00
	*Ae*. *apicoargenteus*	4	1	5	0.07	0	0	0	0.00	0	0	0	0.00
	*Ae*. *argenteopunctatus*	1	1	2	0.03	0	0	2	0.01	0	0	0	0.00
	*Ae*. *dendrophilus*	122	114	236	3.08	5	1	6	0.04	0	0	0	0.00
	*Ae*. *furcifer*	134	145	279	3.64	3	3	6	0.04	0	0	0	0.00
	*Ae*. *haworthi*	0	0	0	0.00	23	28	51	0.30	0	0	0	0.00
	*Ae*. *lilii*	41	33	74	0.97	0	0	0	0.00	0	0	0	0.00
	*Ae*. *longipalpis*	7	4	11	0.14	0	0	0	0.00	0	0	0	0.00
	*Ae*. *luteocpehalus*	49	43	92	1.20	0	0	0	0.00	0	0	0	0.00
	*Ae*. *metallicus*	41	38	79	1.03	7	11	18	0.11	0	0	0	0.00
	*Ae*. *opok*	19	24	43	0.56	0	0	0	0.00	0	0	0	0.00
	*Ae*. *palpalis*	126	118	244	3.18	0	0	0	0.00	0	0	0	0.00
	*Ae*. *stokesi*	0	2	2	0.03	0	0	0	0.00	0	0	0	0.00
	*Ae*. *unilineatus*	41	33	74	0.97	0	0	0	0.00	0	0	0	0.00
	*Ae*. *usambara*	18	23	41	0.53	0	0	0	0.00	0	0	0	0.00
	*Ae*. *vittatus*	91	83	174	2.27	384	359	743	4.39	65	78	143	0.53
	**Total**	**3108**	**3051**	**6159**	**80.40**	**7096**	**7251**	**14347**	**84.75**	**11593**	**11381**	**22974**	**85.19**
***Anopheles***	*An*. *coustani*	1	2	3	0.04	0	0	0	0.00	0	0	0	0.00
	*An*. *gambiae*	41	37	78	1.02	46	37	83	0.49	63	68	131	0.48
	*An*. *pharoensis*	10	7	17	0.22	6	2	8	0.05	1	0	1	0.01
	*An*. *rufipes*	0	1	1	0.01	0	0	0	0.00	0	0	0	0.00
	*An*. *ziemani*	6	7	13	0.17	0	0	0	0.00	0	0	0	0.00
	**Total**	**58**	**54**	**112**	**1.46**	**52**	**39**	**91**	**0.54**	**64**	**68**	**132**	**0.49**
***Coquelitidia***	*Cq*. *cristata*	1	3	4	0.05	0	0	0	0.00	0	0	0	0.00
	*Cq*. *fuscopennata*	3	0	3	0.04	0	0	0	0.00	0	0	0	0.00
	**Total**	**4**	**3**	**7**	**0.09**	**0**	**0**	**0**	**0.00**	**0**	**0**	**0**	**0.00**
***Culex***	*Cx*. *annulioris*	5	2	7	0.09	0	0	0	0.00	0	0	0	0.00
	*Cx*. *cinereus*	48	46	94	1.23	0	0	0	0.00	0	0	0	0.00
	*Cx*. *decens*	12	17	29	0.38	10	13	23	0.14	7	11	18	0.07
	*Cx*. *nebulosus*	56	42	98	1.28	39	34	73	0.42	23	18	41	0.14
	*Cx*. *poicilipes*	137	126	263	3.43	0	0	0	0.00	0	0	0	0.00
	*Cx*. *quinquefasciatus*	321	297	618	8.07	1165	1099	2264	13.37	1761	1873	3634	13.48
	*Cx*. *tigripes*	34	39	73	0.95	59	72	131	0.77	78	91	169	0.63
	**Total**	**613**	**569**	**1182**	**15.43**	**1273**	**1218**	**2491**	**14.70**	**1869**	**1993**	**3862**	**14.32**
***Eretmapodites***	*Er*. *chrysogaster*	57	66	123	1.61	2	0	2	0.01	0	0	0	0.00
	*Er*. *inornatus*	3	5	8	0.10	0	0	0	0.00	0	0	0	0.00
	*Er*. *quinquevittatus*	9	4	13	0.17	0	0	0	0.00	0	0	0	0.00
	**Total**	**69**	**75**	**144**	**1.88**	**2**	**0**	**2**	**0.01**	**0**	**0**	**0**	**0.00**
***Filcabia***	*Fi*. *circumtesta*	0	1	1	0.01	0	0	0	0.00	0	0	0	0.00
	**Total**	**0**	**1**	**1**	**0.01**	**0**	**0**	**0**	**0.00**	**0**	**0**	**0**	**0.00**
***Toxorhynchites***	*Tx*. *brevipalpis*	28	18	46	0.60	0	0	0	0.00	0	0	0	0.00
	*Tx*. *lutescens*	4	3	7	0.09	0	0	0	0.00	0	0	0	0.00
	**Total**	**32**	**21**	**53**	**0.69**	**0**	**0**	**0**	**0.00**	**0**	**0**	**0**	**0.00**
***Uranotenia***	*Ur*. *mashonensis*	2	1	3	0.04	0	0	0	0.00	0	0	0	0.00
	**Total**	**2**	**1**	**3**	**0.04**	**0**	**0**	**0**	**0.00**	**0**	**0**	**0**	**0.00**
**Total**		**3886**	**3775**	**7661**	**100**	**8423**	**8508**	**16931**	**100**	**13526**	**13442**	**26968**	**100**

F, female; M, male; T, total numbers of mosquitoes. %: proportion in percentage (%) of specimens of mosquitoes.

The predacious mosquito species *Cx*. *tigripes* was sampled in each of the three study settings, while the predators *Eretmapodites chrysogaster*, *Er*. *inornatus*, and *Toxorhynchites brevipalpis* were primarily collected in rural settings. Moreover, several other vector competent mosquito species, namely *Anopheles coustani*, *An*. *gambiae*, *Coquelettidia fuscopennata*, *Cx*. *quinquefasciatus*, and *Cx*. *poicilipes* were sampled.

### Ecological characterization of *Aedes* species and breeding sites

Table 3 summarizes the species composition of *Aedes* mosquitoes collected as larvae among different types of breeding sites in the rural, suburban, and urban areas. *Ae*. *aegypti* and *Ae*. *vittatus* were commonly encountered in the three settings. *Ae*. *aegypti* was the most prevalent species in the all study areas, and exhibited rising abundance from rural (n = 6,159; 75.12%) to suburban (n = 14,347, 93.94%), and urban (n = 22,974, 99.37%) areas. The highest prevalence of *Ae*. *vittatus* (5.18%) was found in suburban areas. In rural areas, *Ae*. *furcifer* (4.53%), *Ae*. *palpalis* (3.96%), *Ae*. *dendrophilus* (3.83%), *Ae*. *vittatus* (2.83%), *Ae*. *africanus* (2.31%), *Ae*. *luteocephalus* (1.49%), *Ae*. *metallicus* (1.28%), *Ae*. *lilii* (1.22%), and *Ae*. *unilineatus* (1.20%) were collected at frequencies above 1%. We also found two specimens of *Ae*. *albopictus* (0.01%) in the urban settings.

**Table 3:** Proportions (%) of *Aedes* mosquito species collected as larvae among different types of breeding sites in rural, suburban, and urban areas in south-eastern Côte d'Ivoire from January 2013 to October 2014.

Species	Natural^a^^,^^b^							Artificial^a^													Total
								Traditional^b^				Industrial^b^								Total	
	Rock^c^	Anim^c^	Leaf^c^	Husk^c^	Bamb^c^	Tree^c^	Total	Clay^c^	Wood^c^	Metal^c^	Total	Tarp^c^	Disca^c^	Tire^c^	Tank^c^	Carca^c^	build^c^	Stora^c^	Total		
Rural																					
*Ae*. *aegypti*	0.41	0.47	0.54	4.58	1.27	1.23	8.49	4.34	1.64	3.41	9.38	3.88	12.97	26.04	3.52	6.10	2.24	2.48	57.25	66.63	75.12
*Ae*. *africanus*	0.00	0.00	0.00	0.50	0.70	1.12	2.32	0.00	0.00	0.00	0.00	0.00	0.00	0.00	0.00	0.00	0.00	0.00	0.0	0.00	2.31
*Ae*. *angustus*	0.05	0.00	0.00	0.03	0.00	0.10	0.18	0.00	0.00	0.00	0.00	0.00	0.05	0.31	0.00	0.00	0.00	0.00	0.36	0.36	0.54
*Ae*. *apicoargenteus*	0.00	0.00	0.00	0.00	0.00	0.00	0.00	0.05	0.00	0.00	0.05	0.00	0.03	0.00	0.00	0.00	0.00	0.00	0.03	0.08	0.08
*Ae*. *argenteopunctatus*	0.00	0.00	0.00	0.00	0.00	0.00	0.00	0.00	0.00	0.00	0.00	0.00	0.00	0.03	0.00	0.00	0.00	0.00	0.03	0.03	0.03
*Ae*. *dendrophilus*	0.00	0.13	0.26	0.18	0.15	0.29	1.01	0.05	0.26	0.11	0.42	0.15	0.62	1.56	0.00	0.08	0.00	0.00	2.40	2.83	3.83
*Ae*. *furcifer*	0.32	0.00	0.00	0.00	0.31	0.75	1.38	0.00	0.00	0.00	0.00	0.52	0.18	1.74	0.26	0.45	0.00	0.00	3.15	3.15	4.53
*Ae*. *lilii*	0.00	0.00	0.17	0.60	0.08	0.05	0.90	0.00	0.00	0.00	0.00	0.05	0.05	0.16	0.00	0.06	0.00	0.00	0.32	0.32	1.22
*Ae*. *longipalpis*	0.00	0.00	0.00	0.11	0.05	0.02	0.18	0.00	0.00	0.00	0.00	0.00	0.00	0.00	0.00	0.00	0.00	0.00	0.00	0.00	0.18
*Ae*. *luteocephalus*	0.00	0.05	0.00	0.18	0.00	0.11	0.34	0.00	0.00	0.00	0.00	0.05	0.19	0.68	0.00	0.23	0.00	0.00	1.15	1.15	1.49
*Ae*. *metallicus*	0.08	0.00	0.00	0.00	0.00	0.05	0.13	0.47	0.08	0.34	0.89	0.00	0.06	0.19	0.00	0.00	0.00	0.00	0.26	1.15	1.28
*Ae*. *opok*	0.00	0.00	0.00	0.00	0.00	0.05	0.05	0.00	0.00	0.00	0.00	0.03	0.00	0.62	0.00	0.00	0.00	0.00	0.65	0.65	0.70
*Ae*. *palpalis*	0.70	0.00	0.06	0.15	0.21	0.71	1.83	0.29	0.15	0.00	0.44	0.29	0.34	0.94	0.00	0.11	0.00	0.00	1.69	2.13	3.96
*Ae*. *stokesi*	0.00	0.00	0.00	0.00	0.00	0.03	0.03	0.00	0.00	0.00	0.00	0.00	0.00	0.00	0.00	0.00	0.00	0.00	0.00	0.00	0.03
*Ae*. *unilineatus*	0.00	0.03	0.00	0.13	0.08	0.28	0.52	0.26	0.05	0.18	0.49	0.00	0.00	0.15	0.00	0.05	0.00	0.00	0.19	0.68	1.20
*Ae*. *usambara*	0.00	0.00	0.00	0.00	0.42	0.24	0.67	0.00	0.00	0.00	0.00	0.00	0.00	0.00	0.00	0.00	0.00	0.00	0.00	0.00	0.67
*Ae*. *vittatus*	0.21	0.00	0.00	0.00	0.10	0.39	0.70	0.10	0.23	0.15	0.47	0.44	0.34	0.62	0.08	0.06	0.11	0.00	1.66	2.13	2.83
**Total**	**1.77**	**0.68**	**1.03**	**6.46**	**3.37**	**5.42**	**18.7**	**5.56**	**2.41**	**4.19**	**12.14**	**5.41**	**14.83**	**33.04**	**3.86**	**7.14**	**2.35**	**2.48**	**69.14**	**81.29**	**100**
**Suburban**																					
*Ae*. *aegypti*	na	0.06	0.00	0.47	0.24	0.00	0.77	2.47	1.15	2.52	6.14	2.83	26.21	42.70	2.10	8.21	1.92	3.06	87.03	**93.17**	93.94
*Ae*. *angustus*	na	0.00	0.00	0.00	0.00	0.00	0.00	0.09	0.00	0.06	0.15	0.00	0.05	0.10	0.00	0.00	0.00	0.00	0.15	**0.30**	0.30
*Ae*. *argenteopunstatus*	na	0.00	0.00	0.00	0.00	0.01	0.01	0.00	0.00	0.00	0.00	0.00	0.00	0.00	0.00	0.00	0.00	0.00	0.00	**0.00**	0.01
*Ae*. *dendrophilus*	na	0.00	0.01	0.00	0.00	0.00	0.01	0.00	0.00	0.00	0.00	0.00	0.00	0.03	0.00	0.00	0.00	0.00	0.03	**0.03**	0.04
*Ae*. *furcifer*	na	0.00	0.00	0.04	0.00	0.00	0.04	0.00	0.00	0.00	0.00	0.00	0.00	0.00	0.00	0.00	0.00	0.00	0.00	**0.00**	0.04
*Ae*. *haworthi*	na	0.00	0.00	0.00	0.00	0.02	0.02	0.16	0.01	0.00	0.17	0.08	0.00	0.07	0.00	0.01	0.00	0.00	0.16	**0.33**	0.36
*Ae*. *metallicus*	na	0.00	0.00	0.00	0.00	0.00	0.00	0.00	0.02	0.08	0.10	0.00	0.00	0.02	0.00	0.00	0.00	0.00	0.02	**0.13**	0.13
*Ae*. *vittatus*	na	0.08	0.00	0.04	0.15	0.00	0.27	0.53	0.27	0.78	1.58	0.62	0.47	1.81	0.03	0.00	0.30	0.09	3.32	**4.91**	5.18
**Total**	na	**0.14**	**0.01**	**0.5**	**0.39**	**0.03**	**1.12**	**3.25**	**1.45**	**3.44**	**8.14**	**3.53**	**26.73**	**44.73**	**2.13**	**8.22**	**2.22**	**3.15**	**90.71**	**98.87**	**100**
**Urban**																					
*Ae*. *aegypti*	na	0.00	0.00	0.18	0.13	na	0.32	0.45	0.41	1.11	1.97	1.97	24.49	60.14	2.67	3.83	0.67	3.32	97.08	**99.05**	99.37
*Ae*. *albopictus*	na	0.00	0.00	0.00	0.00	na	0.00	0.00	0.00	0.00	0.00	0.00	0.00	0.01	0.00	0.00	0.00	0.00	0.01	**0.01**	0.01
*Ae*. *vittatus*	na	0.00	0.00	0.03	0.05	na	0.08	0.10	0.00	0.00	0.10	0.24	0.03	0.15	0.00	0.00	0.00	0.02	0.44	**0.54**	0.62
**Total**	**na**	**0.00**	**0.00**	**0.21**	**0.18**	na	**0.40**	**0.55**	**0.41**	**1.11**	**2.07**	**2.21**	**24.52**	**60.30**	**2.67**	**3.83**	**0.67**	**3.34**	**97.53**	**99.60**	**100**

%: percentage

^a,b,c^: The inspected containers were grouped into the two categories^a^, three sub-categories^b^, and 16 types^c^, as indicated above. The container type often reflects the name of the container. The total numbers of specimens of *Aedes* mosquitoes collected were 6,159, 14,347, and 22,974 specimens in the rural, suburban, and urban areas, respectively. Rock, rock holes; Anim, animal detritus; Husk, fruit husks; Bamb, bamboo holes; Tree, tree holes; Clay, clay pots; Wood, wood-containers; Metal, metallic pots; Disca, discarded containers; Tank, vehicle tanks; Carca, vehicle carcasses; Building, Building tool; Stora, water storage containers.

The presence of *Aedes* mosquito larvae in breeding sites significantly varied by species (Table 3). For example, *Ae*. *aegypti* were found in all types of *Aedes*-positive breeding sites sampled in all the three study areas. Moreover, *Ae*. *dendrophilus*, *Ae*. *furcifer*, and *Ae*. *luteocephalus* were found in all container types in the rural areas, while *Ae*. *vittatus* and *Ae*. *metallicus* were collected from both natural and artificial containers in the suburban areas. *Ae*. *africanus*, *Ae*. *lilii*, *Ae*. *unilineatus*, and *Ae*. *usambara* were mostly present in natural containers such as tree holes, bamboo, and fruit husks in rural settings.

### Associations among different *Aedes* species

Several species were found together in the same breeding sites. For example, *Ae*. *aegypti*, *Ae*. *dendrophilus*, *Ae*. *furcifer*, and *Ae*. *africanus* shared the same breeding sites in the rural areas, whereas *Ae*. *aegypti* co-existed with *Ae*. *vittatus* in suburban settings (n = 1,295, 12.8%). These two species co-occurred, albeit at low frequency (n = 57, 0.3%) in urban breeding sites. Additionally, *Cx*. *quinquefasciatus* and *An*. *gambiae* were often collected together with *Ae*. *aegypti* in tires and discarded containers in peri-domestic environments in the three study areas.

Mosquito predators, such as *Cx*. *tigripes*, *Er*. *chrysogaster*, and *Tx*. *brevipalpis* were found in the same breeding sites as *Ae*. *aegypti*, *Ae*. *dendrophilus*, *Ae*. *furcifer*, and *Ae*. *africanus* in rural settings. These ecologic associations were most present in tree holes, discarded containers, and tires in the rural areas and in peri-domestic breeding sites during the rainy season.

### *Aedes* breeding site positivity

Among 3,569, 4,882, and 5,783 containers inspected in rural, suburban, and urban settings, 2,423, 3,069, and 3,374 were wet, respectively. The urban setting had a significantly higher *Aedes*-positive breeding site rate (2,136/3,374, FP = 63.3%), as compared to suburban (1,428/3,069, FP = 46.5%) and rural settings (738/2,423, FP = 30.5%) (χ^2^ = 478.9, df = 2, p < 0.05) (S1 Table). The Mann-Whitney U test indicated that the abundance of immature *Aedes* mosquitoes in one study area was significantly different compared to another. A significantly higher abundance of immature *Aedes* mosquitoes was found in urban areas with larval densities of 1.26 ± 0.01 larvae/l, followed by the suburban areas with 0.77 ± 0.01 larvae/l and rural areas with 0.42 ± 0.01 larvae/l (χ^2^ = 663.3, df = 2, p < 0.001) (Table 4). Urban settings showed significantly higher proportions of pupae (n = 23,126, 14.9%) and 3–4 instar larvae compared to rural setting with 9.6% (n = 6,212) of pupae and 47.8% of 3–4 instar larvae (p < 0.05). The presence of immature *Aedes* mosquitoes was significantly associated with the sites, seasons, breeding site types and categories, substrates, color, vegetal detritus, shade, water turbidity, and predators (p < 0.05).

**Table 4:** *Aedes* mosquito species dynamics, as revealed by larval collections in breeding sites in rural, suburban, and urban areas in south-eastern Côte d’Ivoire from January 2013 to October 2014.

Characteristic	Rural				Suburban				Urban			
	Abundance (mean ± SE)	Richness	Shannon’s diversity index	Simpson’s dominance index	Abundance (mean ± SE)	Richness	Shannon’s diversity index	Simpson’s dominance index	Abundance (mean ± SE)	Richness	Shannon’s diversity index	Simpson’s dominance index
**Areas**	0.42 ± 0.01	17	1.64	0.57	0.77 ± 0.01	8	0.38	0.89	1.26 ± 0.01	3	0.06	0.99
**Site**												
Peri-domestic	0.65 ± 0.02	16	1.69	0.55	1.39 ± 0.02	8	0.39	0.89	2.10 ± 0.01	3	0.04	0.99
Domestic	0.13 ± 0.01	14	1.20	0.70	0.23 ± 0.01	3	0.35	0.88	0.88 ± 0.37	2	0.13	0.97
**Breeding site**												
Rock hole	0.22 ± 0.08	6	2.18	0.26	na	na	na	na	na	na	na	na
Animal detritus	0.12 ± 0.04	4	1.41	0.52	0.78 ± 0.30	2	0.99	0.51	0.00 ± 0.00	0	na	na
Leaf axil	0.10 ± 0.03	4	1.68	0.36	0.06 ± 0.06	1	0.00	1.00	0.00 ± 0.00	0	na	na
Fruit husk	0.44 ± 0.04	9	1.62	0.52	0.71 ± 0.15	3	0.76	0.73	0.67 ± 0.29	2	0.54	0.78
Bamboo	0.34 ± 0.06	10	2.64	0.22	0.51 ± 0.21	2	0.96	0.53	0.78 ± 0.26	2	0.85	0.60
Tree hole	0.98 ± 0.05	15	3.13	0.14	0.21 ± 0.14	2	0.97	0.52	na	na	na	na
**Natural**	**0.32 ± 0.02**	**15**	**2.75**	**0.25**	**0.46 ± 0.18**	**6**	**1.31**	**0.52**	**0.02 ± 0.01**	**2**	**0.72**	**0.68**
Clay pot	0.58 ± 0.06	7	1.23	0.62	0.74 ± 0.07	4	1.09	0.61	0.99 ± 0.12	2	0.70	0.69
Wood	0.50 ± 0.08	6	1.04	0.67	0.63 ± 0.08	4	0.87	0.66	1.06 ± 0.19	1	0.00	1.00
Metallic pot	1.23 ± 0.10	5	1.04	0.67	0.83 ± 0.06	4	1.04	0.59	0.88 ± 0.09	1	0.00	1.00
**Traditional**	**0.67 ± 0.04**	**7**	**1.31**	**0.61**	**0.92 ± 0.07**	**5**	**1.07**	**0.61**	**1.02 ± 0.14**	**2**	**0.29**	**0.90**
Tarp	0.80 ± 0.08	8	1.50	0.53	0.60 ± 0.06	3	0.83	0.67	0.88 ± 0.08	2	0.49	0.81
Discarded	0.73 ± 0.04	10	0.88	0.77	0.99 ± 0.02	3	0.15	0.96	1.83 ± 0.02	2	0.01	0.99
Tire	1.20 ± 0.04	12	1.37	0.63	1.78 ± 0.02	6	0.30	0.91	2.30 ± 0.02	3	0.03	0.99
Vehicle tank	0.63 ± 0.07	3	0.50	0.84	1.45 ± 0.10	2	0.12	0.97	1.93 ± 0.06	1	0.00	1.00
Vehicle carcasses	0.51 ± 0.04	8	0.95	0.73	0.89 ± 0.04	2	0.01	0.99	1.36 ± 0.06	1	0.00	1.00
Building tools	0.70 ± 0.11	2	0.28	0.91	1.01 ± 0.08	2	0.57	0.77	0.91 ± 0.10	1	0.00	1.00
Water storage	0.03 ± 0.01	1	0.00	1.00	0.07 ± 0.01	2	0.19	0.94	0.13 ± 0.01	1	0.06	0.99
**Industrial**	**0.80 ± 0.02**	**13**	**1.16**	**0.69**	**1.32 ± 0.01**	**6**	**0.27**	**0.92**	**2.12 ± 0.01**	**3**	**0.04**	**0.99**
**Artificial**	**0.70 ± 0.02**	**13**	**1.23**	**0.68**	**1.17 ± 0.01**	**6**	**0.36**	**0.89**	**2.07 ± 0.01**	**3**	**0.06**	**0.99**

SE, standard error of the mean number of mosquitoes per liter of water. The abundance is expressed as the mean number of *Aedes* mosquito larvae per liter of water (larvae/l) and calculated as Williams’ mean

### Dynamics of *Aedes* breeding sites

**Fig 2:**
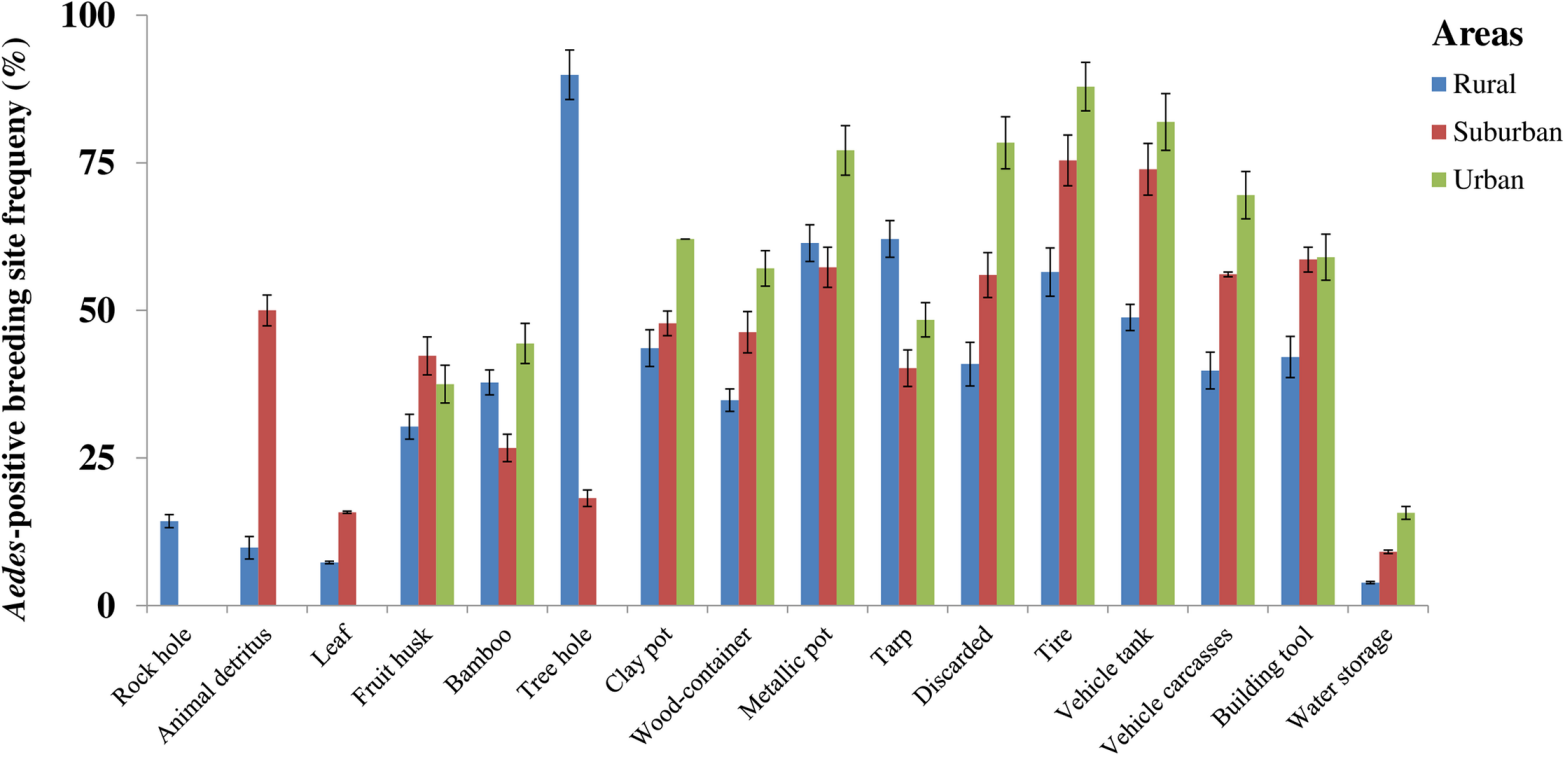
Dynamics of *Aedes* mosquito breeding sites in rural, suburban, and urban areas in south-eastern Côte d’Ivoire from January 2013 to October 2014. Error bars show the standard error (SE).

**Fig 3:**
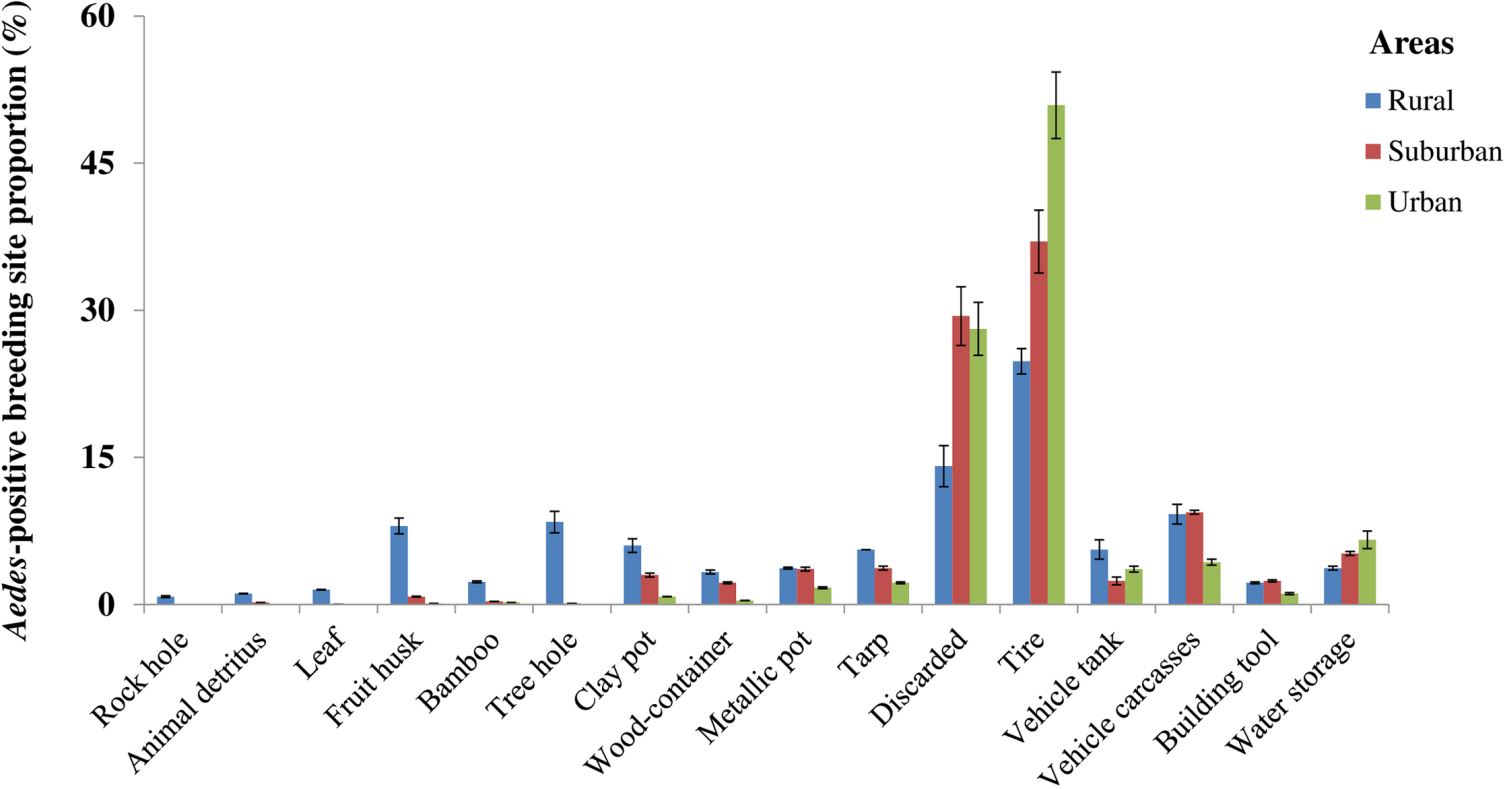
Frequency of *Aedes* mosquito breeding sites in rural, suburban, and urban areas in south-eastern Côte d’Ivoire from January 2013 to October 2014. Error bars show the standard error (SE).

Fig 2 shows that the *Aedes*-positive microhabitat rate varied widely from one breeding site type to another in all three areas. The rural area showed the largest variability in *Aedes* breeding sites grouped into 16 types, followed by the suburban and urban areas presenting 15 and 12 microhabitat types, respectively. S1 Table indicates that immature *Aedes* mosquitoes were found in both natural (163/738, PP = 22.1%) and artificial (575/738, PP = 77.9%) breeding sites in the rural, and mostly in artificial breeding sites in the suburban (1,405/1,428, PP = 98.4%) and urban (2,129/2,136, PP = 99.7%) areas, including higher proportions of industrial containers in the urban areas (2,066/2,136, PP = 96.7%). In the rural areas, the main *Aedes*-positive breeding sites represented natural types, such as three holes (62/69, FP = 89.9%), bamboo (17/45, FP = 37.8%), and fruit husks (59/195, FP = 30.3%), traditional containers such as metallic (27/44, FP = 61.4%) and clay pots (44/101, FP = 43.6%) and wood-containers (24/69, FP = 34.8%); and industrial containers such as tarps (41/66, FP = 62.1%), tires (183/324, FP = 56.5%), vehicle tanks (41/84, FP = 48.8%), discarded containers (104/254, FP = 40.9%), and vehicle carcasses (68/171, FP = 52.0%). In the urban setting, the most common *Aedes* breeding sites comprised of industrial containers such as tires (1,087/1,236, FP = 87.9%), discarded containers (601/767, FP = 78.4%), vehicle tanks (77/94, FP = 81.9%), vehicle carcasses (91/131, FP = 69.5%), and water storage containers (141/896, FP = 15.7%). Water storage containers were found to be more frequently infested with immature stages of *Aedes* mosquitoes in the urban than in the suburban (χ^2^ = 17.3, df = 1, p < 0.001) or rural settings (χ^2^ = 57.3, df = 1, p < 0.001). Furthermore, there was a statistically significant difference in *Aedes* mosquito positivity rate in water storage container between the suburban and rural settings (χ^2^ = 15.8, df = 1, p < 0.001). Besides the variations in the frequency in the colonization of *Aedes* breeding sites, the most abundant *Aedes* breeding sites were tires and discarded containers in all the study areas (all p < 0.05) (Fig 3). Also frequently positive were natural breeding sites such as tree holes (62/738, PP = 8.4%), fruit husks (59/738, PP = 8.0%), industrial containers such as tarps (41/738, PP = 5.6%), vehicle tanks (41/738, PP = 5.6%), and vehicle carcasses (68/738, PP = 9.2%) in the rural area, and water storage containers (141/2,136, PP = 6.6%) in the urban area (Fig 3).

### Ecological variations in *Aedes* species

Table 4 summarizes the abundance, richness, diversity, and dominance of *Aedes* mosquito species according to the breeding site types among sites and study areas. The Shannon’s diversity and Simpson’s dominance indices highly varied between the study areas and breeding sites, showing higher overall values in peri-domestic environments. The highest larval abundances of *Aedes* mosquitoes were recorded in tires in all study areas (p < 0.05). In addition, tree holes and metallic pots in the rural, vehicle tanks and building tools in the suburban, and discarded containers, vehicle tanks, and vehicle carcasses in the urban areas were also highly productive breeding sites for *Aedes* mosquito (S2 Fig). *Aedes* species richness was significantly different among the microhabitats in the rural (F = 4.3, df = 16, p < 0.001), suburban (F = 9.2, df = 7, p < 0.001), and urban settings (F = 11.1, df = 2, p < 0.001). Significantly higher numbers of species (13 species) were found in tree holes in the rural areas. The rural areas showed the highest species diversity, as demonstrated by a Shannon’s diversity index of 1.64, followed by 0.38 for the suburban and 0.06 for the urban areas. Among the breeding sites, the highest Shannon’s diversity index was found in the rural areas for the tree holes with a value of 3.13. Conversely, Simpson’s dominance index of *Aedes* species significantly decreased from the urban (0.99) to suburban (0.89) and rural (0.57) areas (F = 16.2, df = 3, p < 0.001).

### Geographic shifts in *Aedes* breeding sites

Table 5 shows that the proportion of breeding sites positive for *Aedes* larvae significantly varied across the peri-domestic and domestic sites in all study areas. Overall, compared to domestic environment, peri-domestic sites showed a higher proportion of significantly *Aedes-*positive breeding sites, with FP of 84.8% (1,753/2,066) in urban (χ^2^ = 1,100, df = 1, p < 0.001), 70.2% (1,176/1,676) in suburban (χ^2^ = 829.2, df = 1, p < 0.001), and 42.6% (636/1,492) in rural (χ^2^ = 271.5, df = 1, p < 0.001) areas. In rural areas, 87.7% (143/163) of the natural breeding sites that hosted *Aedes* larvae were located in the peri-domestic sites. High numbers of tires were found infested in the domestic site, with FP of 66.5% (151/227) *Aedes*-positive breeding sites in the urban, and 35.8% (63/176) in the suburban area.

**Table 5:** Geographical variations in *Aedes* mosquito breeding site positivity across the sites in the rural, suburban, and urban areas in south-eastern Côte d’Ivoire from January 2013 to October 2014.

Breeding site	Rural								Suburban								Urban							
	Peri-domestic				Domestic				Peri-domestic				Domestic				Peri-domestic				Domestic			
	N	n	FP	PP	N	n	FP	PP	N	n	FP	PP	N	n	FP	PP	N	n	FP	PP	N	n	FP	PP
**Natural**																								
Rock hole	42	6	14.3	0.9	0	0	na	0.0	0	0	na	0.0	0	0	na	0.0	0	0	na	0	0	0	na	0.0
Animal detritus	76	8	10.5	1.3	6	0	0	0.0	6	3	50.0	0.3	0	0	na	0.0	2	0	0	0	0	0	na	0.0
Leaf axil	151	11	7.3	1.7	0	0	na	0.0	19	3	15.8	0.3	0	0	na	0.0	6	0	0	0	0	0	na	0.0
Fruit husk	166	49	29.5	7.7	29	10	34.5	9.8	13	9	69.2	0.8	13	2	15.4	0.8	8	3	37.5	0.2	0	0	na	0.0
Bamboo	45	17	37.8	2.7	0	0	na	0.0	15	4	26.7	0.3	0	0	na	0.0	9	4	44.4	0.2	0	0	na	0.0
Tree hole	56	52	92.9	8.2	13	10	76.9	9.8	11	2	18.2	0.2	0	0	na	0.0	0	0	na	0	0	0	na	0.0
**Total**	**536**	**143**	**26.7**	**22.5**	**48**	**20**	**41.7**	**19.6**	**64**	**21**	**32.8**	**1.8**	**13**	**2**	**15.4**	**0.8**	**25**	**7**	**28**	**0.4**	**0**	**0**	**na**	**0.0**
**Traditional**																								
Clay pot	73	36	49.3	5.7	28	8	28.6	7.8	64	34	53.1	2.9	26	9	34.6	3.6	21	14	66.7	0.8	8	4	50.0	1.0
Wood	42	21	50.0	3.3	27	3	11.1	2.9	41	20	48.8	1.7	26	11	42.31	4.4	11	7	63.6	0.4	3	1	33.3	0.3
Metallic pot	32	22	68.8	3.5	12	5	41.7	4.9	72	43	59.7	3.7	33	18	54.5	7.1	36	30	83.3	1.7	12	7	58.3	1.8
**Total**	**147**	**79**	**53.7**	**12.4**	**67**	**16**	**23.9**	**15.7**	**177**	**97**	**54.8**	**8.2**	**85**	**38**	**44.7**	**15.1**	**68**	**51**	**75**	**2.9**	**23**	**12**	**52.2**	**3.1**
**Industrial**																								
Tarp	39	27	69.2	4.2	27	14	51.9	13.7	47	38	80.9	3.2	85	15	17.6	6.0	58	32	55.2	1.8	37	14	37.8	3.7
Discarded	213	96	45.1	15.1	41	8	19.5	7.8	578	385	66.6	32.7	167	32	19.2	12.7	691	563	81.5	32.1	76	38	50.0	9.9
Tire	286	172	60.1	27.0	38	11	28.9	10.8	520	462	88.8	39.3	176	63	35.8	25.0	1009	936	92.8	53.4	227	151	66.5	39.4
Vehicle tank	81	40	49.4	6.3	3	1	33.3	1.0	46	34	73.9	2.9	0	0	na	0	91	76	83.5	4.3	3	1	33.3	0.3
Carcasses	167	68	40.7	10.7	4	0	0.0	0.0	224	125	55.8	10.6	13	8	61.5	3.2	124	88	71.0	5.0	7	3	42.9	0.8
Building tool	15	9	60.0	1.4	23	7	30.4	6.9	20	14	70.0	1.2	38	20	52.6	7.9	0	0	na	0.0	39	23	59.0	6.0
Water storage	8	2	25.0	0.3	680	25	3.7	24.5	0	0	na	0.0	816	74	9.1	29.4	0	0	na	0.0	896	141	15.7	36.8
**Total**	**809**	**414**	**51.2**	**65.1**	**816**	**66**	**8.1**	**64.7**	**1435**	**1058**	**73.7**	**90.0**	**1295**	**212**	**16.4**	**84.1**	**1973**	**1695**	**85.9**	**96.7**	**1285**	**371**	**28.9**	**96.9**
**Artificial**	**956**	**493**	**51.6**	**77.5**	**883**	**82**	**9.3**	**80.4**	**1612**	**1155**	**71.7**	**98.2**	**1380**	**250**	**18.1**	**99.2**	**2041**	**1746**	**85.5**	**99.6**	**1308**	**383**	**29.3**	**100**
**TOTAL**	**1492**	**636**	**42.6**	**100**	**931**	**102**	**11.0**	**100**	**1676**	**1176**	**70.2**	**100**	**1393**	**252**	**18.1**	**100**	**2066**	**1753**	**84.8**	**100**	**1308**	**383**	**29.3**	**100**

N, number of wet containers; n, number of *Aedes*-positive breeding sites; FP, frequency of *Aedes*-positive breeding sites among wet containers; PP, proportion of each *Aedes*-positive breeding site type among the all *Aedes*-positive breeding site types. The units of FP and PP are percentage (%).

### Seasonal shifts in *Aedes* breeding sites

**Fig 4:**
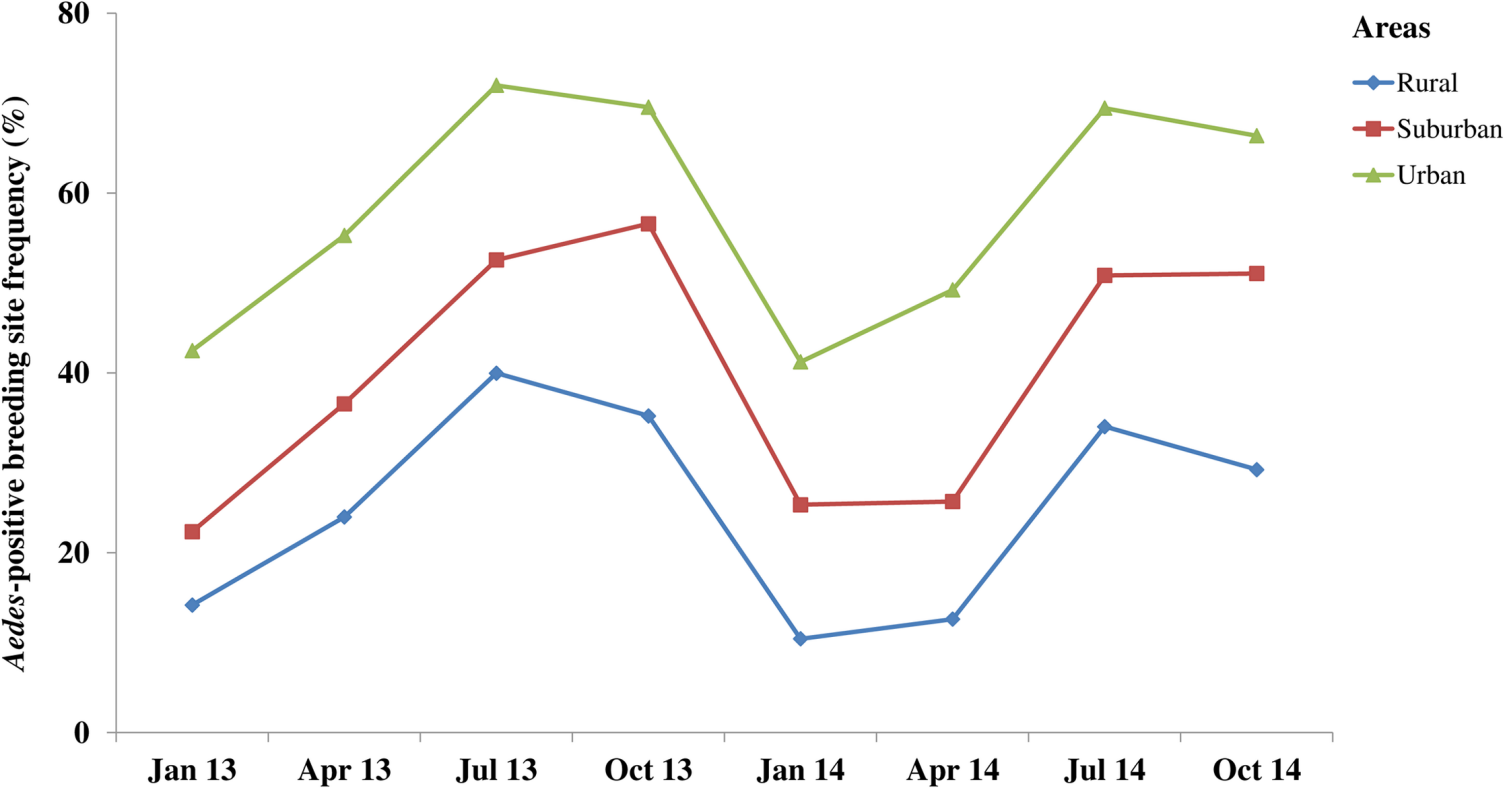
Three-monthly variations in the occurrence of immature stages of *Aedes* mosquitoes in rural, suburban, and urban areas in south-eastern Côte d’Ivoire from January 2013 to October 2014.

**Fig 5:**
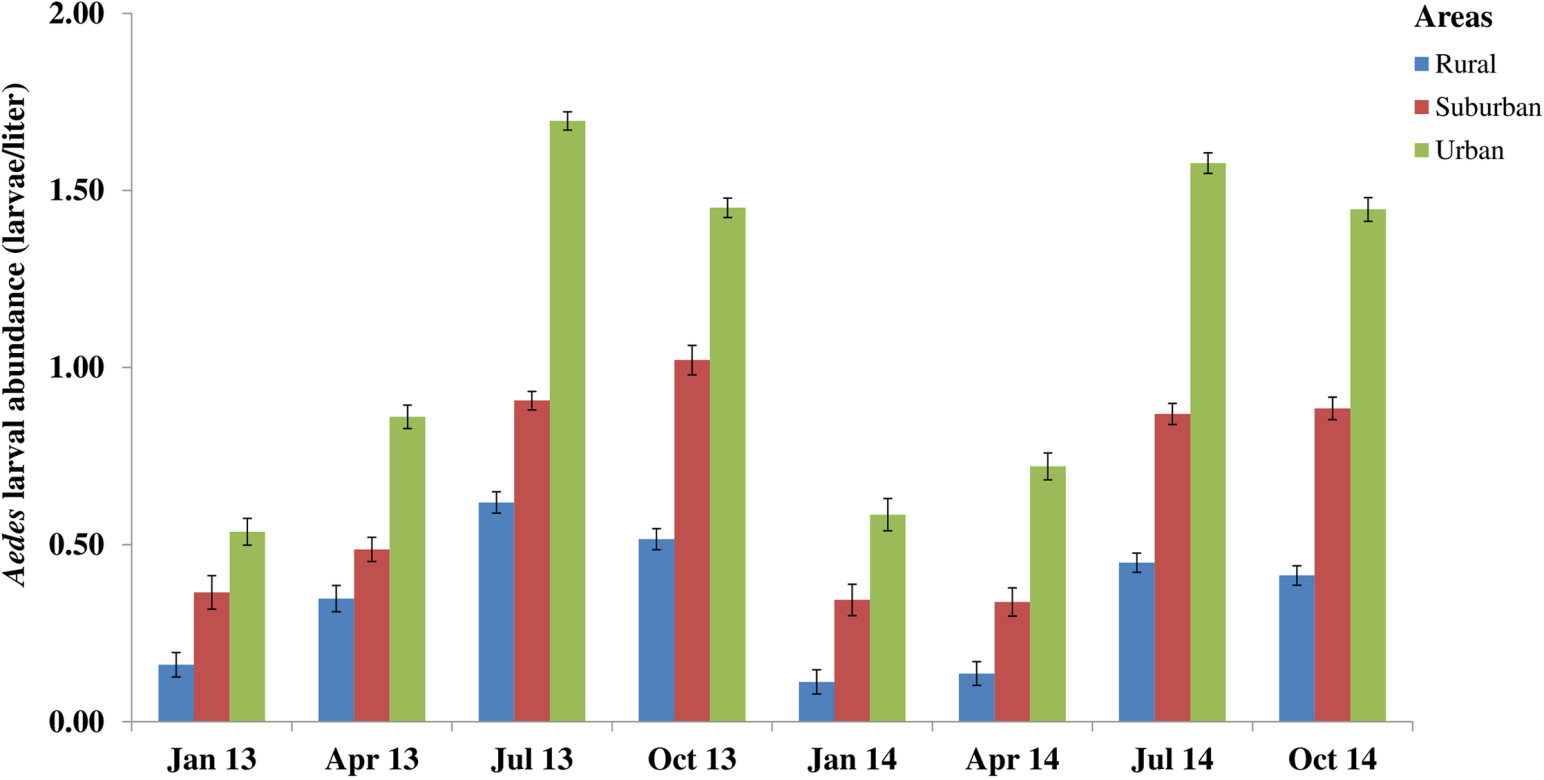
Three-monthly variations in the abundance of immature stages of *Aedes* mosquitoes in rural, suburban, and urban areas in south-eastern Côte d’Ivoire from January 2013 to October 2014. Error bars show the standard error (SE).

In all study areas, the proportion of *Aedes*-positive breeding sites and the number of larvae varied significantly over time with more breeding sites being positive during the rainy season (Fig 4 and S3 Fig). During the rainy season, proportionally more breeding sites were positive. The frequencies of *Aedes*-positive breeding sites were 69.6% (1,650/2,369) in the urban (χ^2^ = 137.7, df = 1, p < 0.001), 52.9% (1,196/2,263) in the suburban (χ^2^ = 138.4, df = 1, p < 0.001), and 34.6% (642/1,857) in the rural (χ^2^ = 63.5, df = 1, p < 0.001) areas (S2 Table). Significantly more *Aedes*-positive breeding sites were observed during the rainy season in the rural, urban, and suburban areas, with FP of 40.0% (187/468) and 72.0% (521/724) in July 2013, and 56.6% (327/578) in October 2013, respectively (S3 Fig). Moreover, higher densities of immature *Aedes* mosquitoes were recorded in July 2013 with 0.62 ± 0.03 and 1.70 ± 0.03 larvae/l in the rural, urban and suburban areas, respectively, and in October 2013 with 1.02 ± 0.02 larvae/l (Fig 5). There were significant differences in the highest *Aedes* microhabitat rates (χ^2^ = 121.2, df = 2, p < 0.001) and the highest abundance (χ^2^ = 156.5, df = 2, p < 0.001) between the three study areas. The highest frequency (i.e., 352/393, FP = 89.6%) of *Aedes*-positive breeding sites was observed in the peri-domestic sites in the urban areas during the rainy season in October 2013.

## Discussion

**Fig 6:**
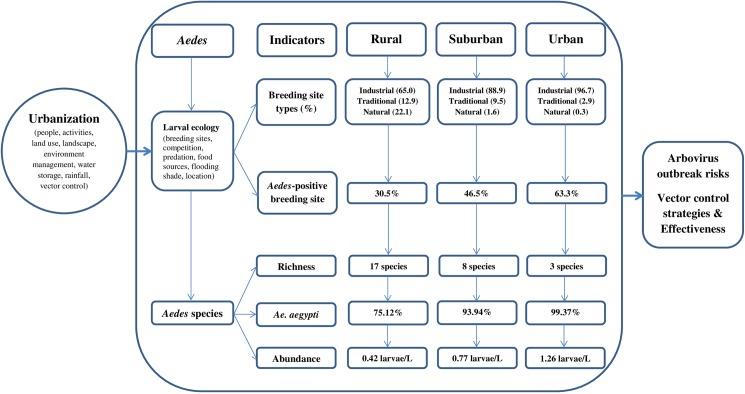
Synthesis of how urbanization shapes immature *Aedes* mosquito breeding sites and species in south-eastern Côte d’Ivoire.

When designing strategies to monitor and control *Aedes* arbovirus vectors in their breeding sites, failure to identify the broad spectrum of potentially available breeding sites will bias the results from field sampling and will thus negatively affect the impact of larval control interventions. Our study pertaining to larval habitats of *Aedes* mosquitoes alongside a rural-to-urban gradient within yellow fever and dengue co-endemic areas in the south-eastern part of Côte d’Ivoire provided strong evidence for influence on species structure, breeding sites, and biological interactions among the immature forms (Fig 6).

Compared to a previous study conducted in the same area of Côte d’Ivoire [16], the current study identified 11 additional *Aedes* species (i.e., *Ae*. *albopictus*, *Ae*. *angustus*, *Ae*. *apicoargentus*, *Ae*. *argenteopunctatus*, *Ae*. *haworthi*, *Ae*. *lilii*, *Ae*. *longipalpis*, *Ae*. *opok*, *Ae*. *palpalis*, *Ae*. *stokesi*, and *Ae*. *unilineatus*) and 16 additional non-*Aedes* species that may influence arbovirus transmission patterns. To our knowledge, *Aedes* mosquito species such as *Ae*. *lilii*, *Ae*. *stokesi*, and *Ae*. *unilineatus*, and others such as *Cq*. *fuscopennata* and *Tx*. *brevipalpis* appear to be reported for the first time in Côte d’Ivoire. *Ae*. *albopictus* is not native to Côte d’Ivoire, but has previously been reported [22]. Presumably this species has been introduced through the seaport bordering the urban municipality of Treichville. The higher numbers of *Aedes* species is likely due to abundant presence of natural and artificial breeding sites, and their potentials to provide suitable microenvironments. Gravid *Aedes* females select oviposition sites according to their physical, chemical, and biological characteristics [11, 12] and these may change in space and time over the year [16].

The public health relevance of *Aedes* mosquitoes results from their invasiveness and ecologic plasticity, competence for multiple pathogens, potential as bridge vectors due to their opportunistic feeding behavior and adaptation to urban, rural, and forest areas [23]. Almost all of the container-specialist *Aedes* mosquitoes collected as larvae such as *Stegomyia* subgenus, including *Ae*. *aegypti*, *Ae*. *africanus*, *Ae*. *albopictus*, *Ae*. *angustus*, *Ae*. *apicoargenteus*, *Ae*. *luteocephalus*, *Ae*. *metallicus*, *Ae*. *opok*, *Ae*. *vittatus*, *Ae*. *unilineatus*, and *Ae*. *usambara* species, and *Diceromyia* and *Aedimorphus* subgenera comprising respectively *Ae*. *furcifer* and *Ae*. *stokesi* species have been shown to carry and/or to transmit in nature over 24 viruses, including yellow fever, dengue, Zika, chikungunya, and Rift Valley in tropical regions [5, 6]. In addition, *Ae*. *(Aedimorphus) argenteopunctatus* in South Africa [24] and *Ae*. *(Neomelaniconion) palpalis* [25] which show vector competence for Rift Valley fever virus *in vitro* and the other *Aedes* species like *Ae*. *(Stegomyia) dendrophilus*, *Ae*. *(Stegomyia) lilii* and *Ae*. *(Aedimorphus) haworthi* which belong to the same subgenera of species involved in the transmission of the arboviruses thus could be suspected as potential vectors of diseases. Still, *Ae*. *(Finlaya) longipalpis* belonging to the same *Finlaya* subgenus with *Ae*. *niveus* which has been the principal vector of dengue virus in Malaysia [26] may potentially transmit arboviruses in Côte d’Ivoire. Among non-*Aedes* mosquitoes, *Er*. *chrysogaster*, *Er*. *inornatus* and *Cq*. *fuscopennata* have been found to have natural infection, while *Er*. *quinquevittatus* has exhibited laboratory competence with yellow fever virus in Africa [6]. Moreover, *An*. *coustani* has been found to be infested with Zika virus [27], while O’nyong-nyong and chikungunya viruses have been isolated from *An*. *gambiae* [28]. *Cx*. *quinquefasciatus* [25] and *Cx*. *poicilipes* [26] have been shown susceptible to transmit Rift Valley fever virus. In conclusion, as in Senegal [12], the collections of immature stages of non-anthropophagic, unexpected and new potential vectors in rural areas suggest the co-existence of several still unidentified arbovirus cycles in south-eastern Côte d’Ivoire.

Our results also revealed that, urban areas showed higher capacity to support *Aedes* breeding sites and larvae than suburban and rural areas. The higher numbers of positive breeding sites and higher abundance of *Aedes* mosquito larvae may be due to the destruction of natural vegetation coverage for infrastructure buildings in the urbanized areas that may affect biological factors (e.g., fauna and flora), and increase the radiation budget thus modifying the microenvironments within and around the microhabitats [29]. Increased exposure to sunlight probably accelerates *Aedes* mosquito larval development and thus increases the size of adult vectors that possibly find more opportunities of blood feeding sources from larger human populations in urban areas [16, 29]. Still, urban *Aedes* populations are probably less exposed to the pressures from agricultural insecticide and predators (e.g., *Eretmapodites* spp. and *Toxorhynchites* spp.) compared to rural communities. We also found that less than two-thirds of breeding sites were infested with *Aedes* larvae thus suggesting that not all available containers filled with water were occupied by at least one larva or pupa of *Aedes* mosquitoes and the immature *Aedes* mosquitoes were not randomly distributed [12]. The presence of empty containers might imply that the gravid females of *Aedes* mosquitoes select their egg-laying sites carefully according to their physical characteristics (e.g., depth, color, clearance, surface, location, height, shade, sun exposure, and food sources) [12, 29], and biological interactions (e.g., competition and predation) [10, 11, 30] at play within the water-holding container systems.

In our larval surveys, we documented distinct geographic and seasonal variations in terms of the proportions of positive breeding sites and abundance of *Aedes* mosquitoes in all areas. Indeed, the highest proportions and relative abundance of *Aedes* mosquitoes were observed among vegetated peri-domestic breeding sites and during the rainy seasons in all areas. The shade of the vegetation reduces the water temperature [12], thus protecting breeding sites from drying out. Moreover, leaves supply organic detritus and associated microorganisms that may serve as food sources for the mosquito larvae [10]. The geographic and seasonal patterns in *Aedes* breeding sites are important from an epidemiologic perspective and suggest that the rainy season is the best period of time to identify breeding sites, while during the dry season it would be an ideal period of time to control immature *Aedes* mosquitoes, with particular attention for peri-domestic environments.

Our data revealed that the pattern of *Aedes* mosquito breeding sites changes substantially from natural containers to artificial containers along a rural-to-urban gradient. Although artificial breeding sites dominate in all areas, there is a higher proportion of natural containers (e.g., rock holes, animal detritus, leaf axils, fruit husks, bamboo, and tree holes) in rural areas, traditional containers (e.g., clay pots, wood-containers, and metallic pots) in suburban areas. However, in the urban areas, the most productive breeding sites for *Aedes* mosquito were industrial containers (e.g., tarps, discarded tires, vehicle tanks, carcasses, building tools, and water storage containers). The availability of, and the segregation among, *Aedes* breeding sites probably result from the strong impacts of human activities on the environment, while the natural breeding sites are provided by the natural landscape and agriculture [12]. We observed that tree holes, tires, and water storage containers showed higher *Aedes* species richness in rural, higher *Aedes* abundances in all areas, and high *Ae*. *aegypti* infestation rates in urban areas, respectively. Tree holes, found in the preserved rainforest, seem to provide ideal larval habitats for several species due to their greater stability, various trophic inputs, and retention of rainwater for longer periods of time [12]. Used tires are mostly associated with the palm oil industry in rural areas, production of the local dish “*Attiéké*” in suburban areas, and selling of tires and car repairs in urban areas. Tree holes and tires have bigger volumes and are expected to better protect the immature forms of *Aedes* mosquitoes against flushing during heavy rains [12, 14]. Moreover, tires are black-colored containers that are highly attractive to the gravid *Aedes* females searching for oviposition sites [11, 31]. The high number of water barrels infested with *Aedes* larvae might be due to the water being held for longer periods in uncovered receptacles [32].

Taken together, *Aedes* species diversity, richness, abundance, and dominance significantly changed from rural to urban settings. The variations in *Aedes* mosquito species may be explained by the sensitivity of their larvae to environmental changes induced by urbanization [10, 12]. Native species such as *Ae*. *africanus*, *Ae*. *argenteopunctatus*, *Ae*. *longipalpis*, *Ae*. *stokesi* and *Ae*. *usambara* were restricted to natural breeding sites in the rural areas. However, other wild species, such as *Ae*. *furcifer*, *Ae*. *dendrophilus*, *Ae*. *palpalis*, *Ae*. *vittatus*, *Ae*. *luteocephalus*, and *Ae*. *metallicus* were also surprisingly frequent in artificial containers. In contrast, our surveillance failed to sample *Ae*. *fraseri* that have been collected by ovitraps in the rural areas previously [16], probably due to its possible cryptic breeding sites or potential height-dependent oviposition behavior. The existence of multiple types of behavior in the same *Aedes* mosquito species may indicate the existence of generalist species or sibling strains of individuals from various origins [6, 11] that have experienced different selective urbanization pressures.

Lastly, our study showed that urbanization acts as a series of ecological filters for *Aedes* mosquitoes by advantaging *Ae*. *aegypti*, the primary vector of yellow fever, dengue, chikunguya, and Zika viruses [1–3]. *Ae*. *aegypti* was the most prevalent species in all study areas, exhibiting an increasing abundance along rural-to-urban gradient towards an higher abundance in urban areas where larvae mostly inhabit in anthropogenic containers (e.g., tires, discarded containers). *Ae*. *aegypti* displayed behavioral plasticity in that the females lay eggs in a vast array of containers ranging from natural containers such as rock holes, tree holes, and bamboo to a wide range of man-made containers [11], including water storage containers in urban areas [32]. The ecologic variations in oviposition behavior of *Ae*. *aegypti* and other *Aedes* mosquitoes may be discussed in ecologic, evolutionary, and epidemiologic approaches [11], and suggest possible overlaps of sylvan and urban vector distributions thus linking several potential mixed arbovirus transmission cycles [5, 6, 12, 16]. In addition, if highly infested microhabitats are targeted for removal, *Aedes* mosquito females may possibly adapt to changes in breeding habitats and alternatively oviposit in other containers previously unoccupied [33]. The ability of *Ae*. *aegypti* to adapt ovipositional behaviors to changing environments possibly enabling to overcome ecological constraints (e.g., instability and disturbance of the breeding sites) imposed by urbanization [10, 11]. *Ae*. *aegypti*-transmitted yellow fever outbreaks are historically well known in Côte d’Ivoire to have forced the transfer of the capital from Grand-Bassam to Abidjan in 1899 [15]. Since then, several unpredictable resurgences of yellow fever and dengue have been occurring in rural and urban areas causing many suspected, confirmed and fatal cases, and remain presently an unresolved major public health concern [7, 15, 34], with the current outbreak of dengue DENV-3 resulting in one confirmed and 17 suspected cases recorded in Abidjan in May 2017. Our study suggests that the unique removal of artificial containers that is a common practice in arbovirus control programs in Côte d’Ivoire might not effectively control diseases in the south-eastern part of the country. Vector control measures should combine removals of artificial containers [6] and autocidal gravid ovitrap-based on mass trapping [35], and insecticide auto-dissemination approaches [36].

## Conclusions

In south-eastern Côte d’Ivoire, urbanization is associated with larval habitats of *Aedes* species at a finer scale by driving their breeding sites from natural to artificial containers, and at the larger scale by transforming rural to urban areas. *Ae*. *aegypti* is most prevalent in urban areas, suggesting that urbanization is a driver for producing suitable breeding sites for this mosquito species, and hence related disease outbreaks. However, rural settings still support irremovable containers such as natural breeding sites (e.g., tree holes) that host several wild *Aedes* species and *Ae*. *aegypti*. Therefore, even effective removal of discarded containers in urban areas (a common practice in arbovirus control programs) might not be sufficient to control arboviral diseases. Instead, vector control strategies should embrace a more holistic approach, combining different tools and methods of proven efficacy [6, 35, 36].

## Supporting information

S1 Fig Range of *Aedes* mosquito breeding sites surveyed in rural, suburban, and urban areas in south-eastern Côte d’Ivoire from January 2013 to October 2014. The container type often reflects the name of the container and the categories include containers that provide comparable larval habitats as follows: A: rock hole, B: animal detritus, C: leaf, D: fruit husks, E: bamboo, F: tree hole, G: clay pot, H: wood-container, I: metallic pot, J: traps, K: discarded container, L: tire, M: vehicle tank, N: vehicle carcasses, O: building tool, P: water storage container.
(PDF)

S2 Fig Variations in abundance of *Aedes* mosquito among breeding sites in rural, suburban, and urban areas in south-eastern Côte d’Ivoire from January 2013 to October 2014. Error bars show the standard error (SE).
(TIF)

S3 Fig Three-monthly variations in the proportions of *Aedes*-positive breeding sites in rural, suburban, and urban areas in south-eastern Côte d’Ivoire from January 2013 to October 2014. Error bars show the standard error (SE).
(TIF)

S1 Table Dynamics of *Aedes* mosquito breeding sites in rural, suburban, and urban areas in south-eastern Côte d’Ivoire from January 2013 to October 2014. (DOCX)

S2 Table Seasonal variations in *Aedes* mosquito breeding site positivity and proportions of positive breeding sites in the rural, suburban and urban areas in south-eastern Côte d’Ivoire from January 2013 to October 2014. (DOCX)
