# Supplementary figures and images for: Urbanization is a main driver for the larval ecology of Aedes mosquitoes in arbovirus-endemic settings in south-eastern Côte d'Ivoire
Source: PLoS Negl Trop Dis. 2017 Jul 13;11(7):e0005751. doi: 10.1371/journal.pntd.0005751 (PMC5526600; doi:10.1371/journal.pntd.0005751)

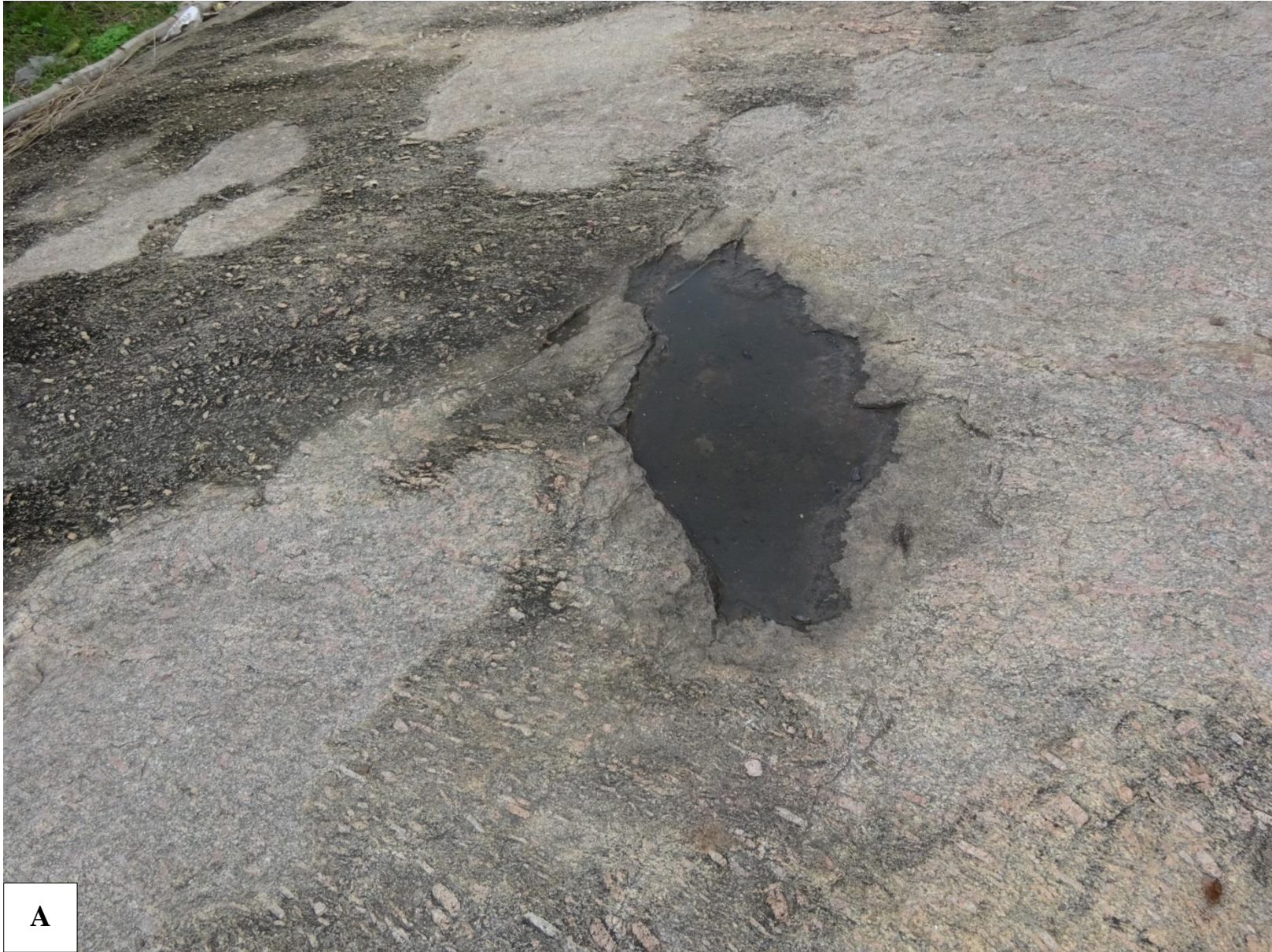

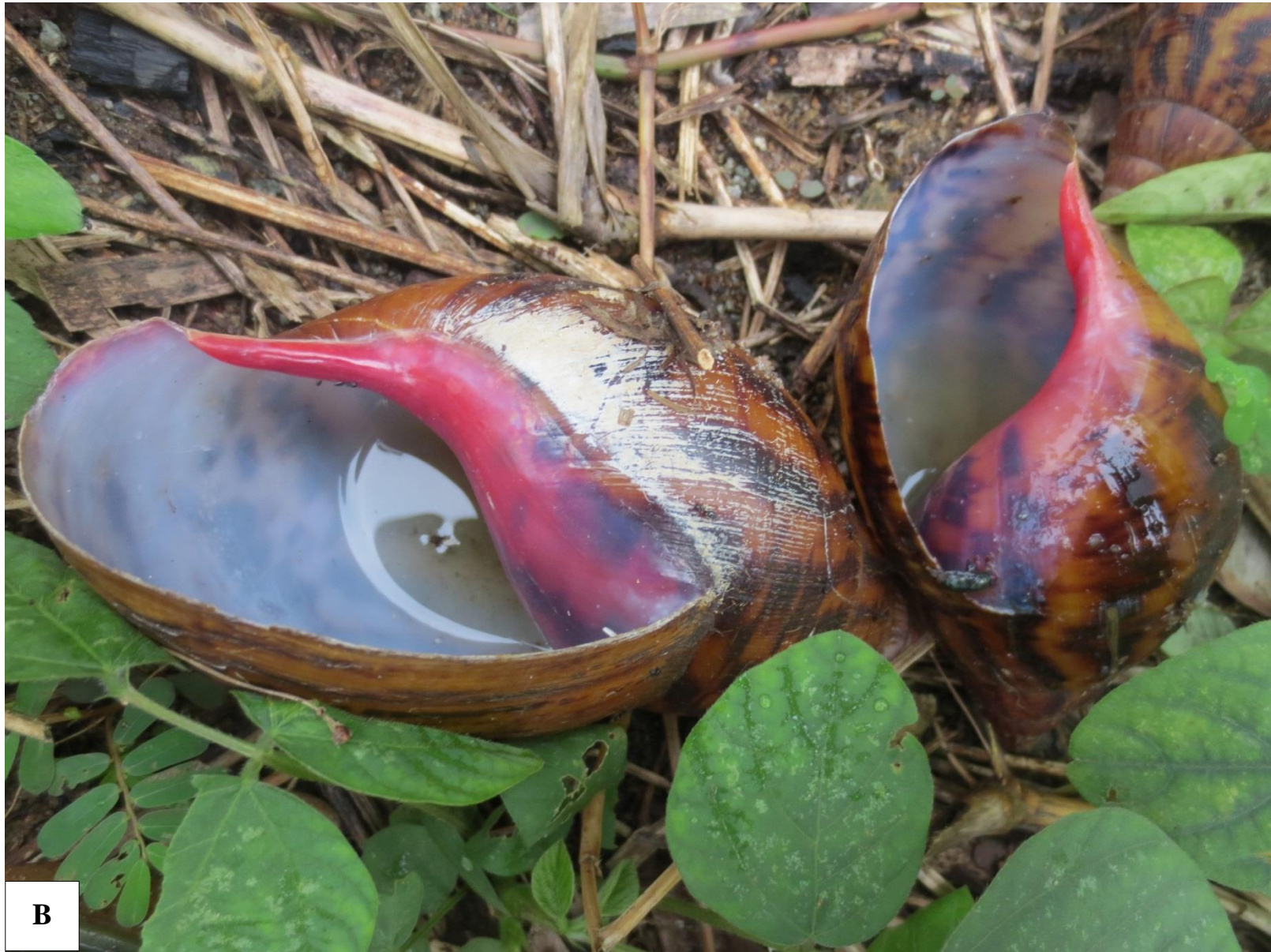

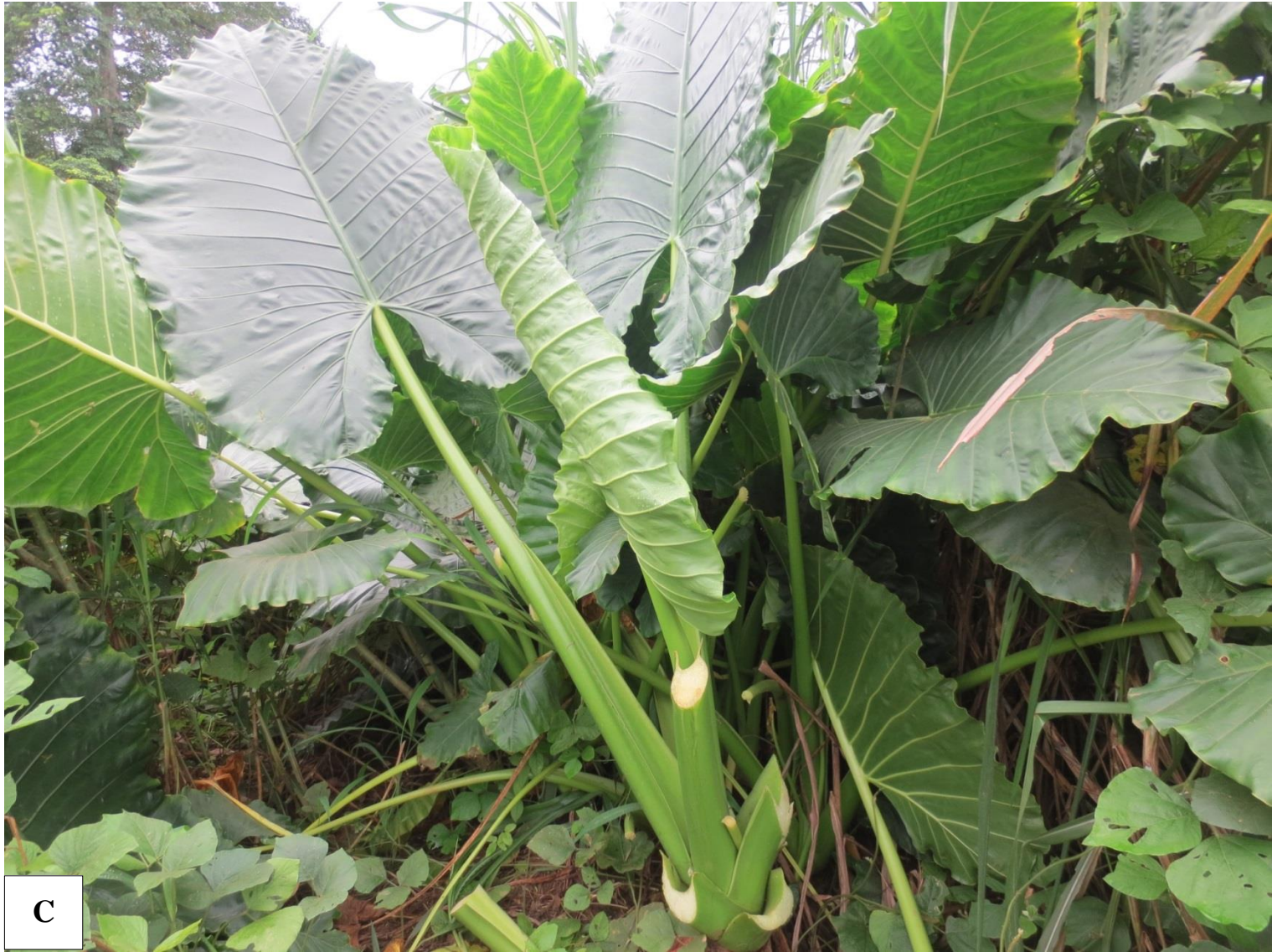

C

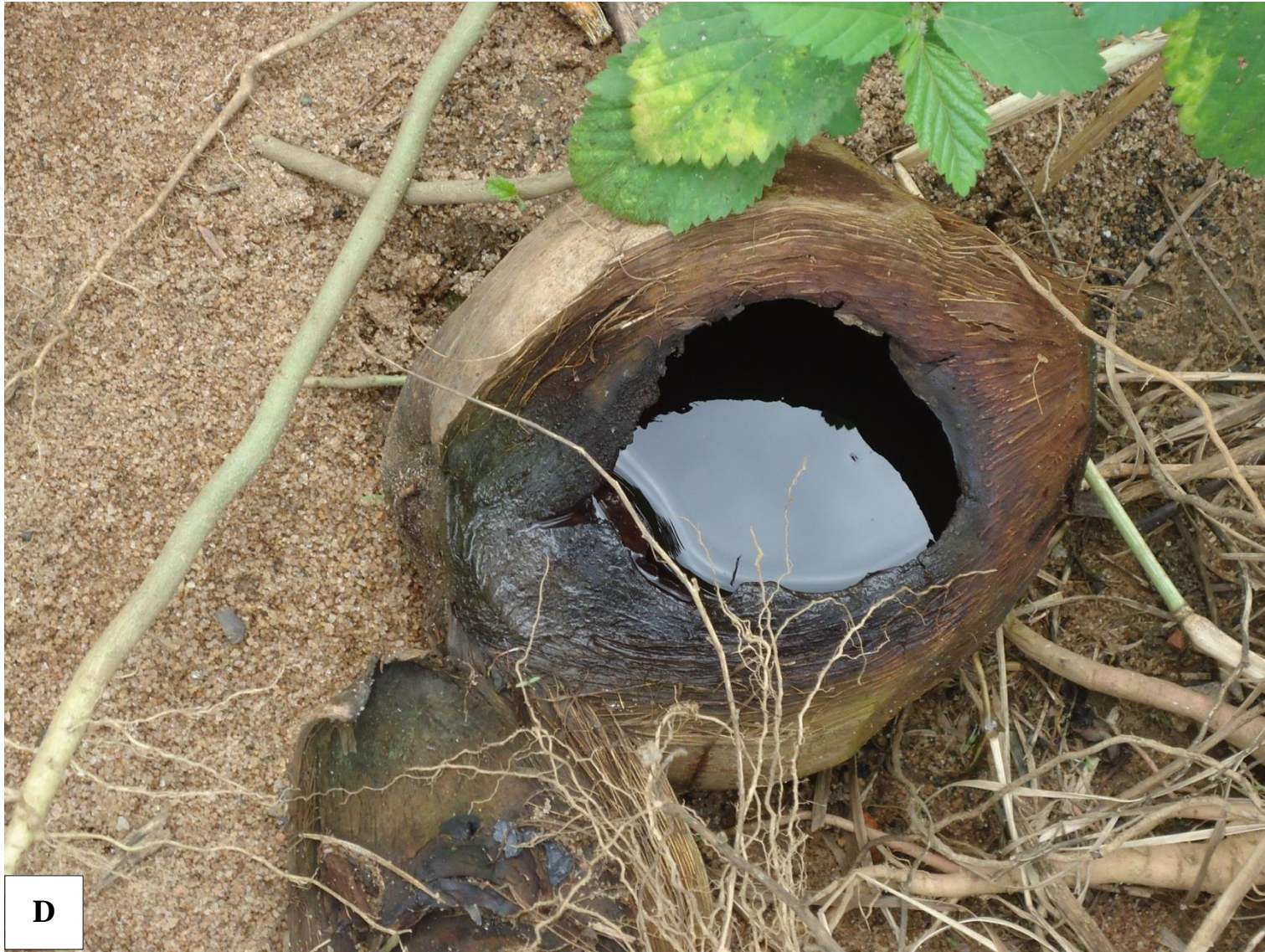

**D**

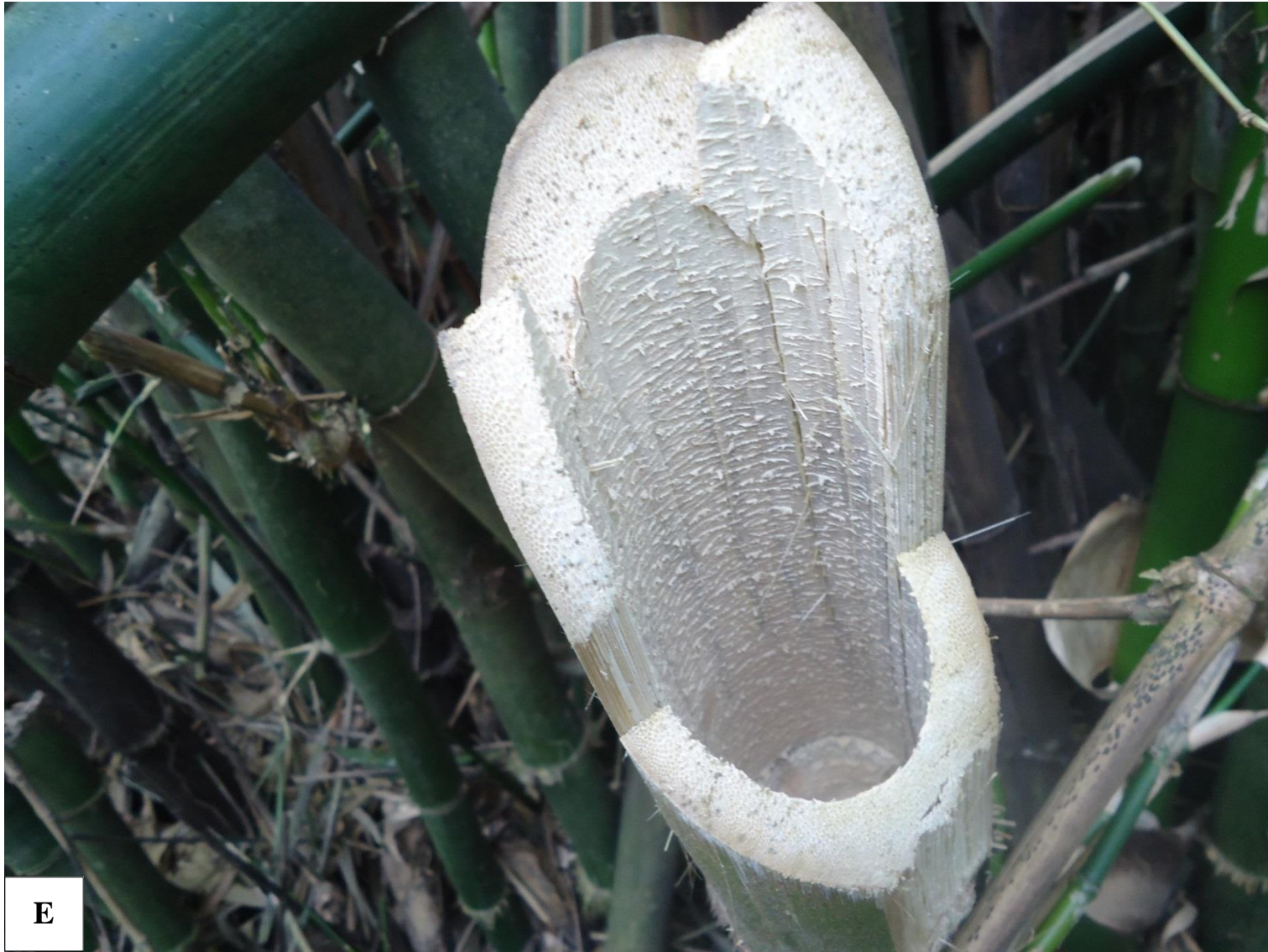

**E**

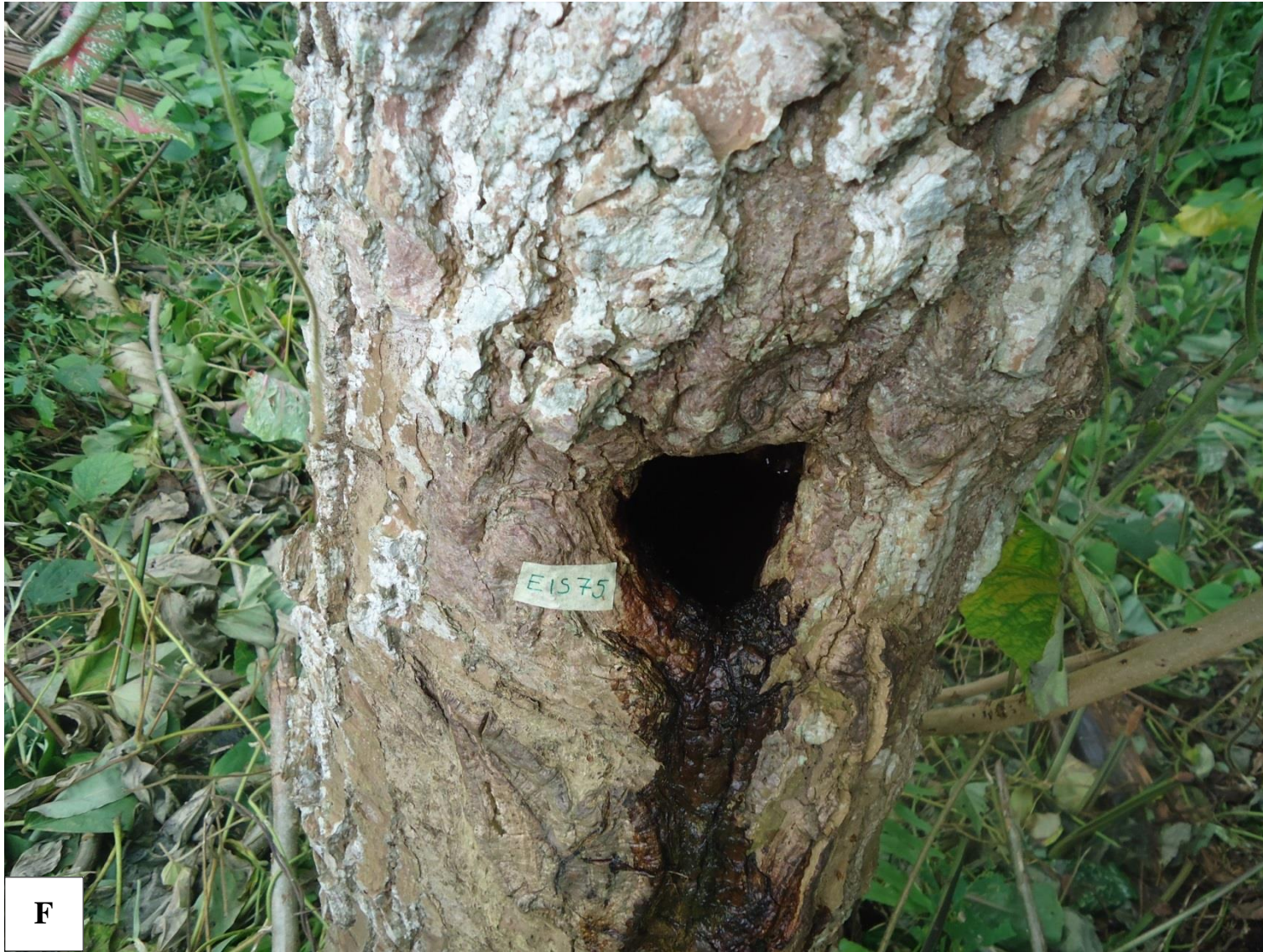

**F**

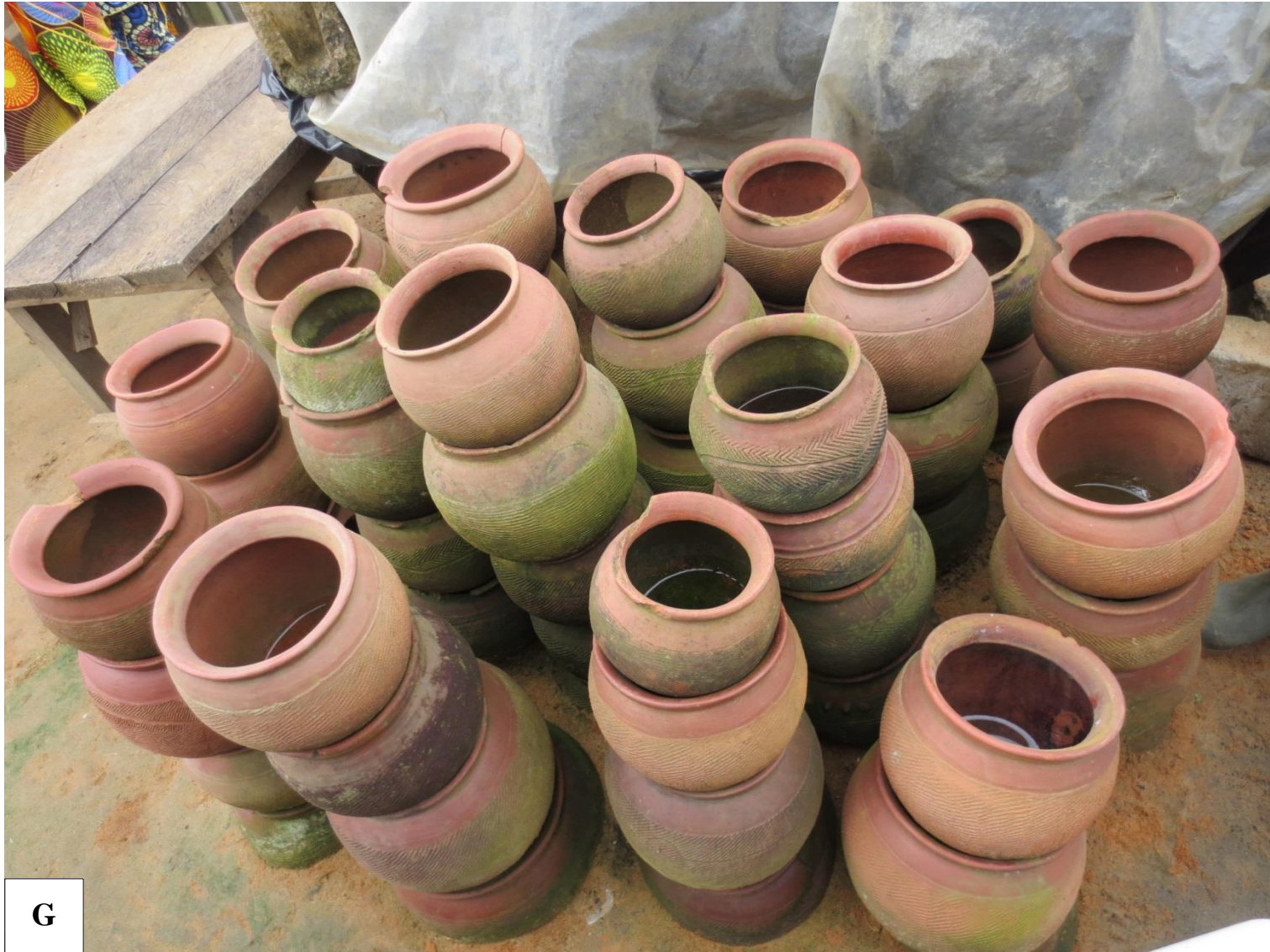

G

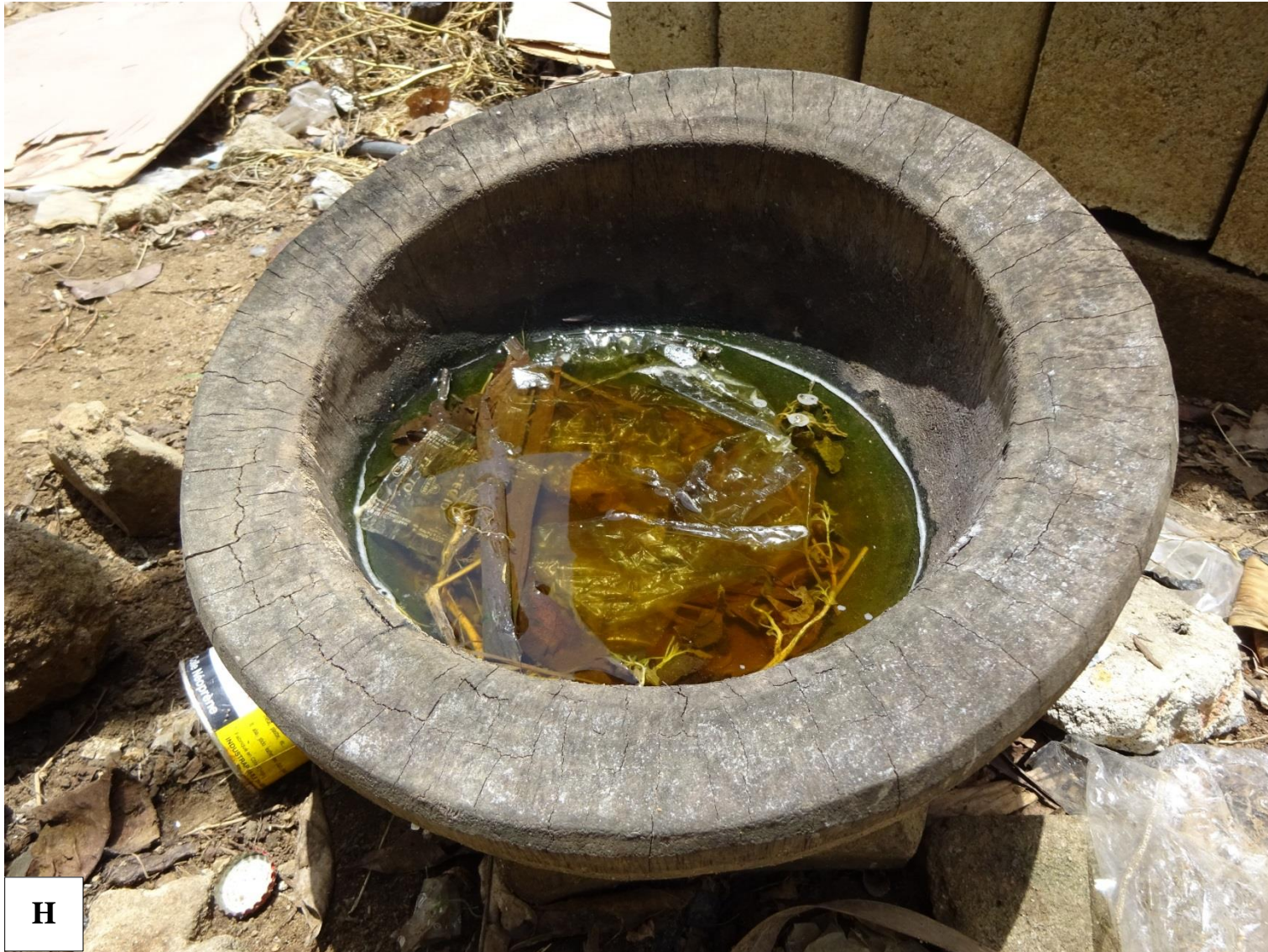

H

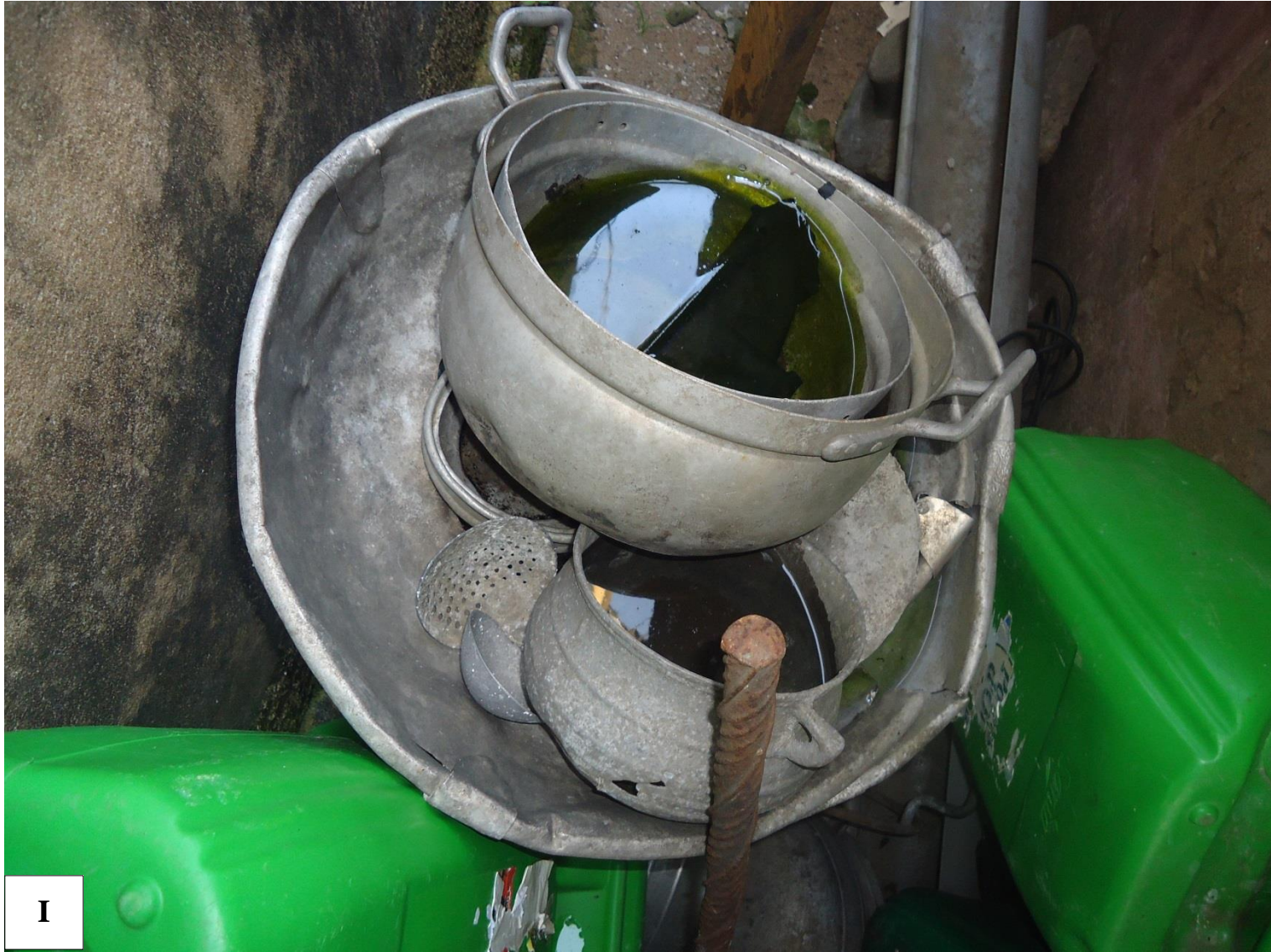

I

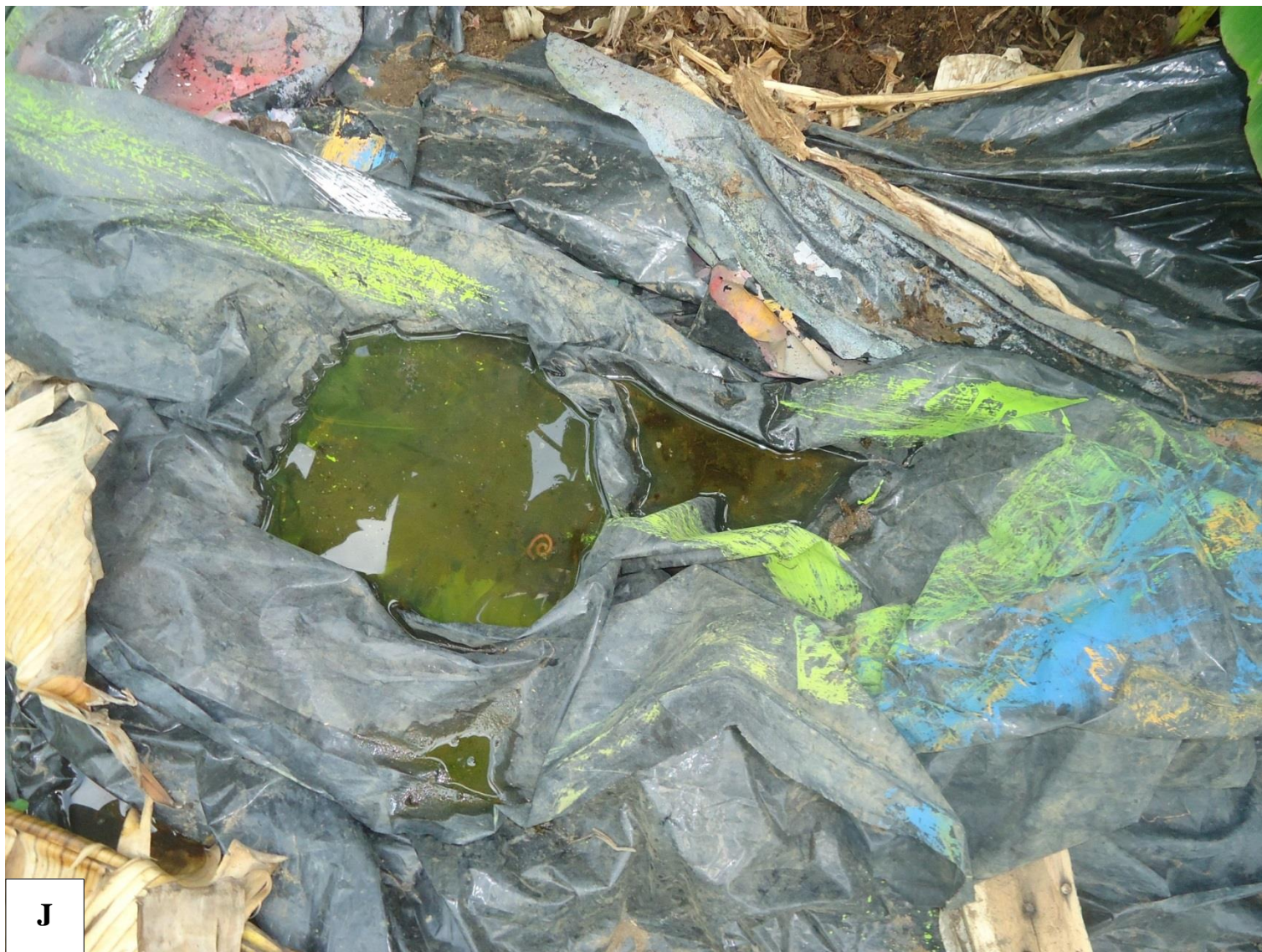

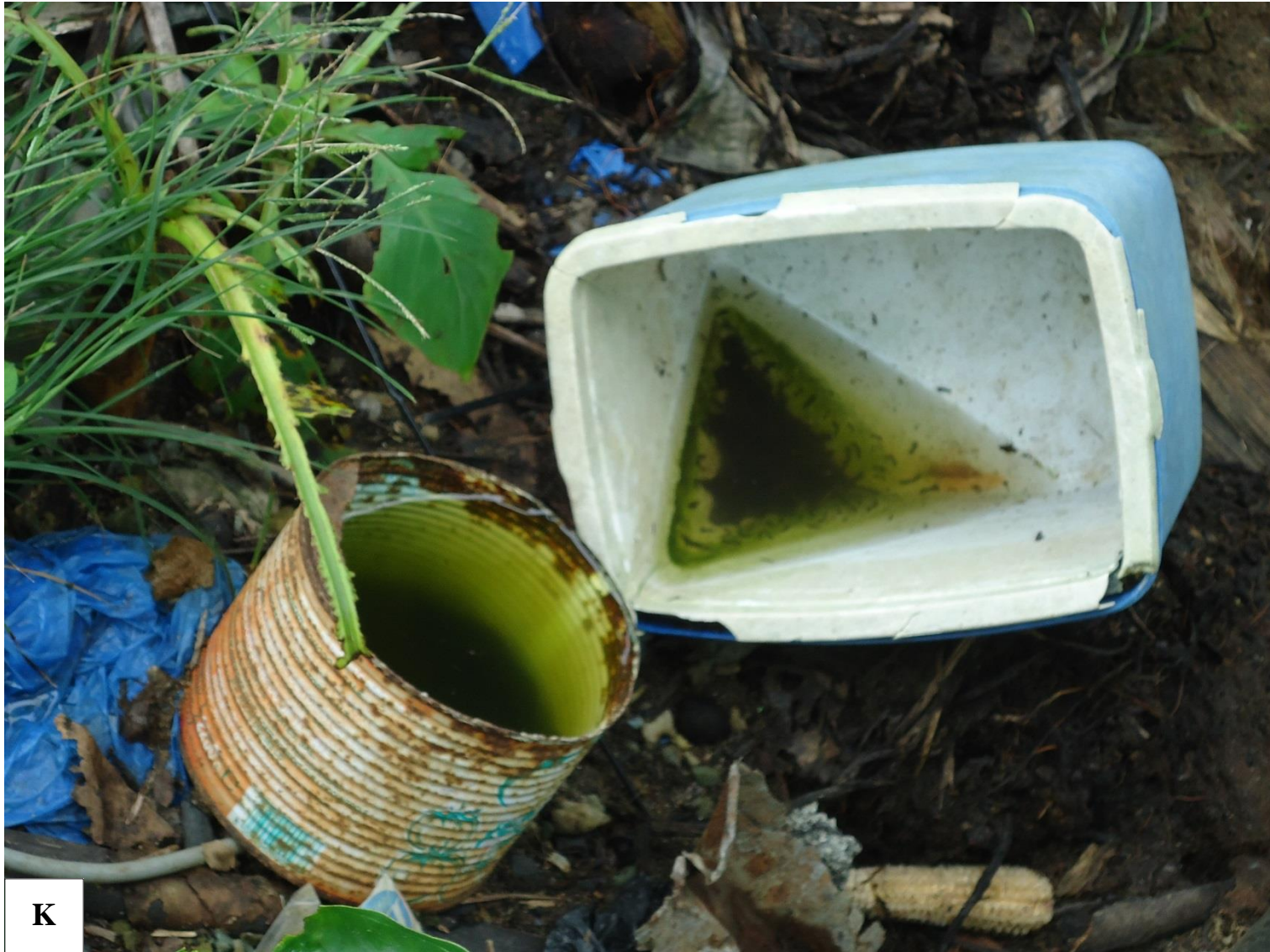

K

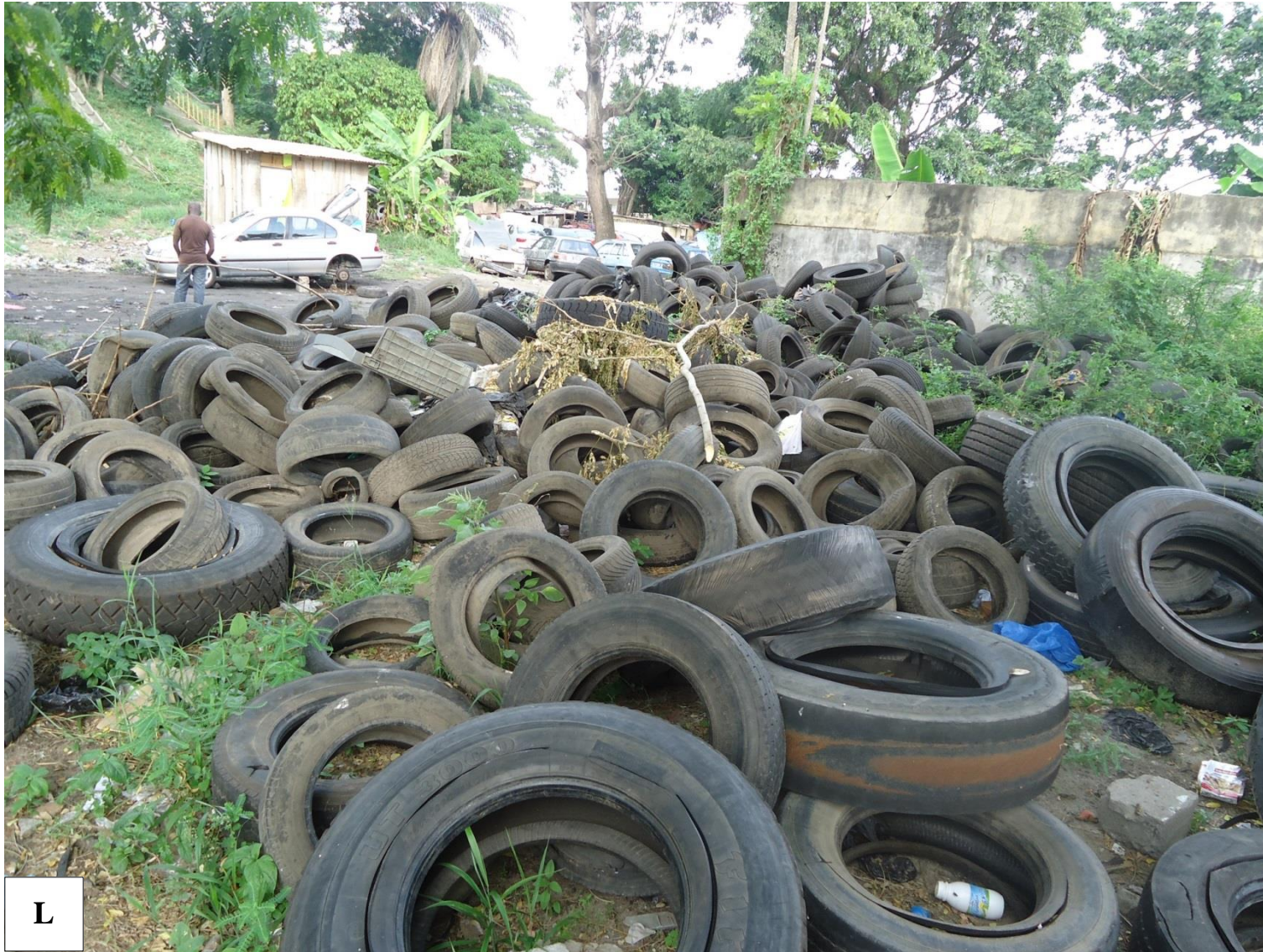

L

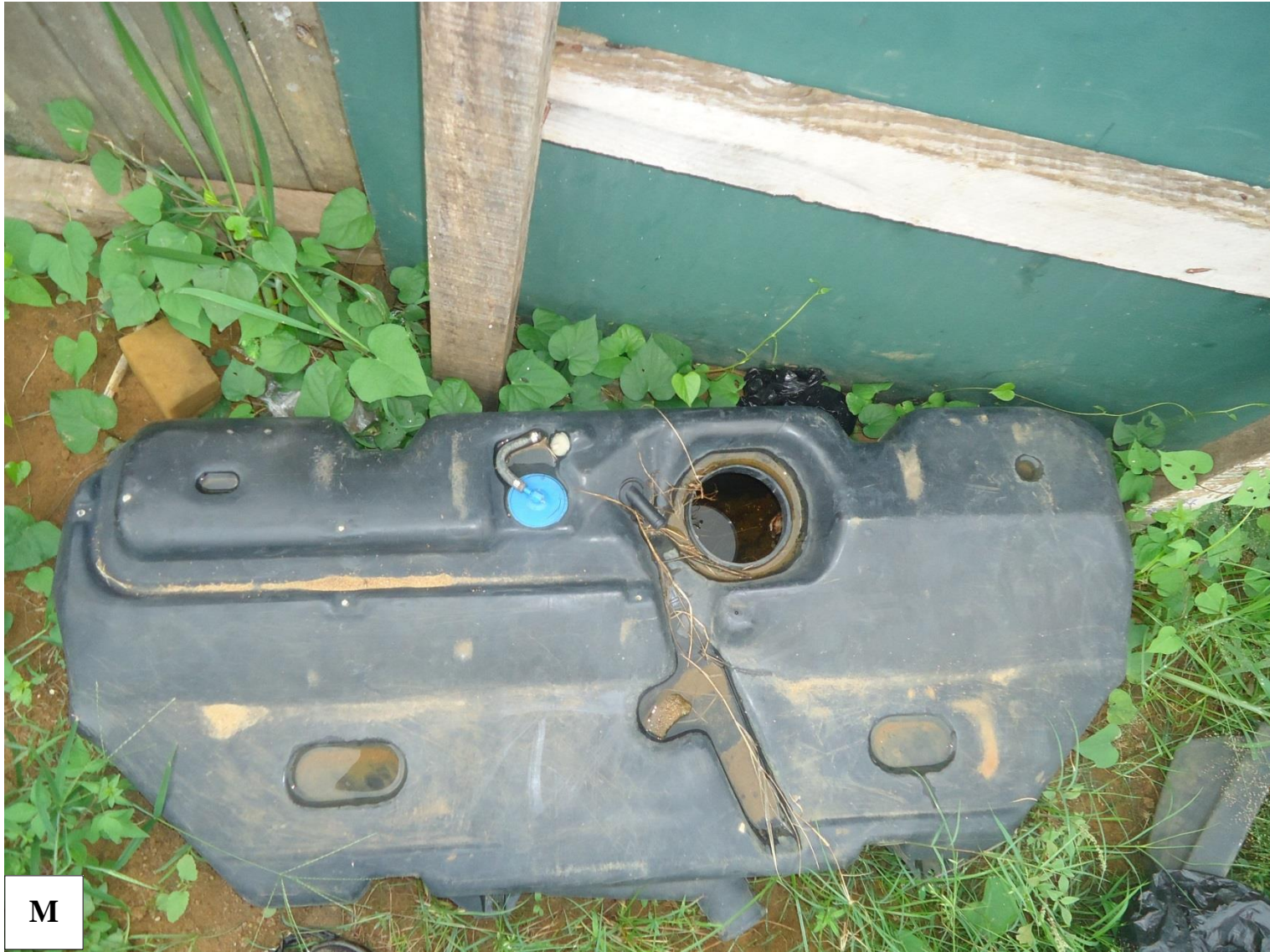

M

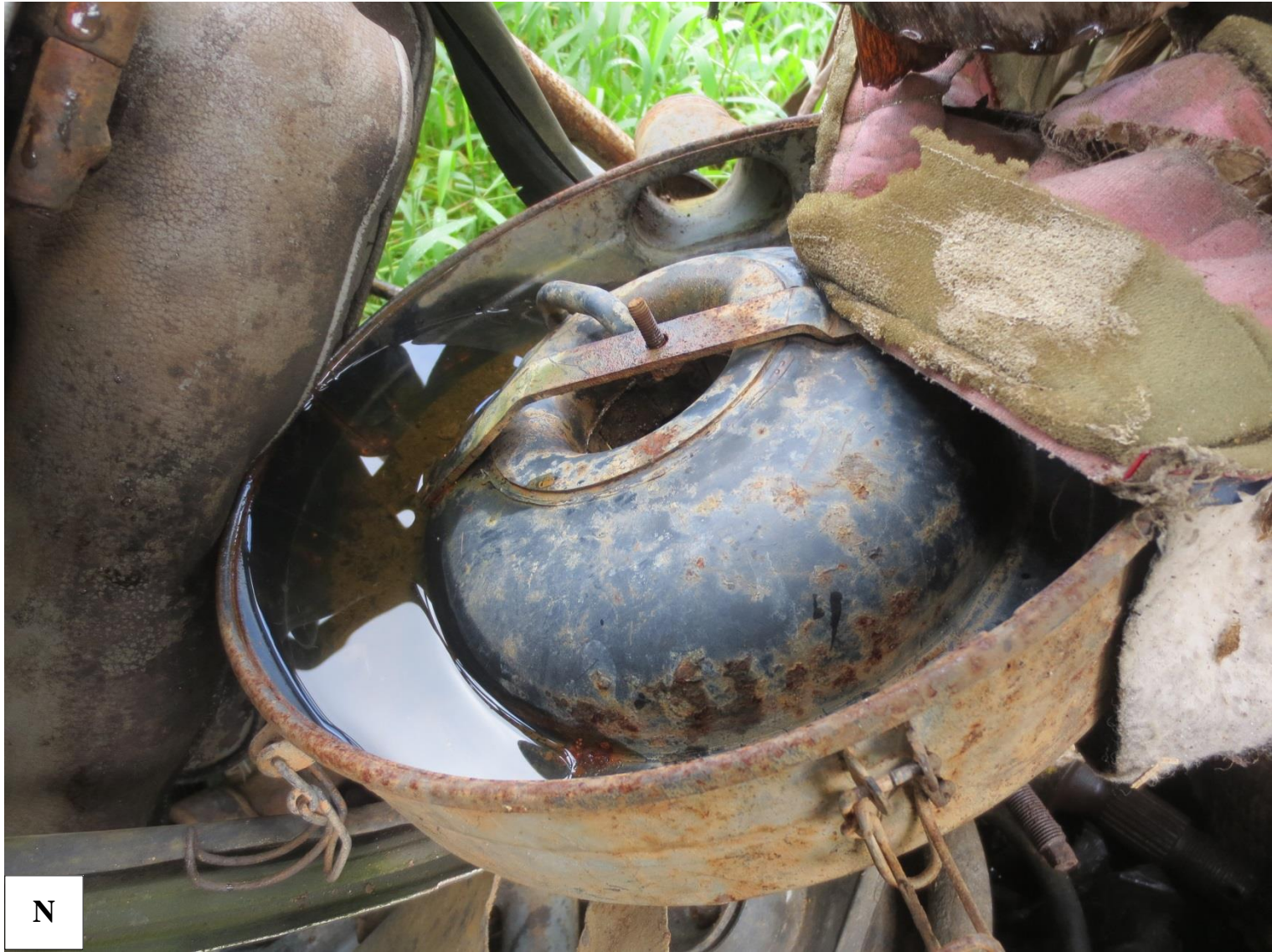

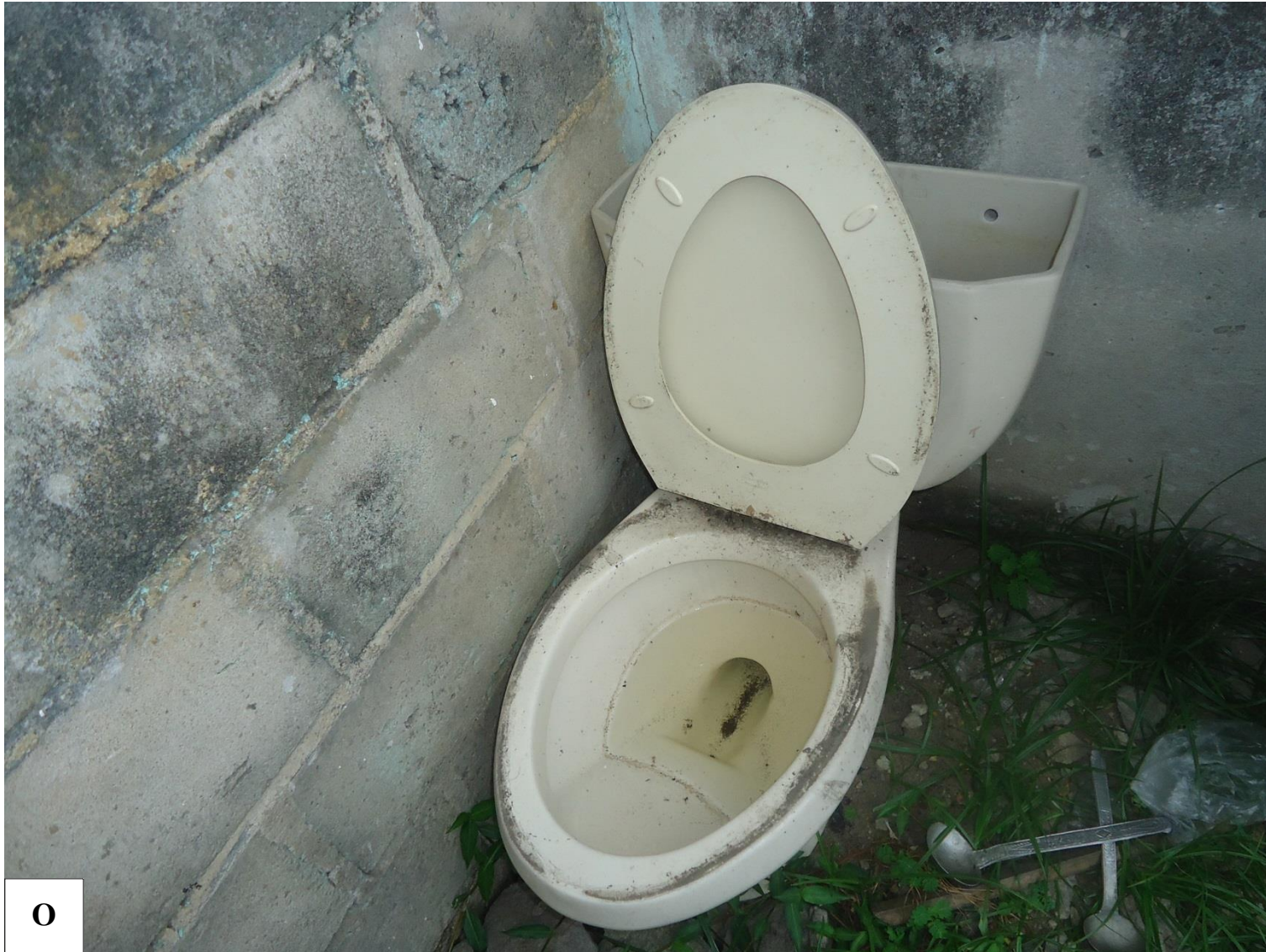

0

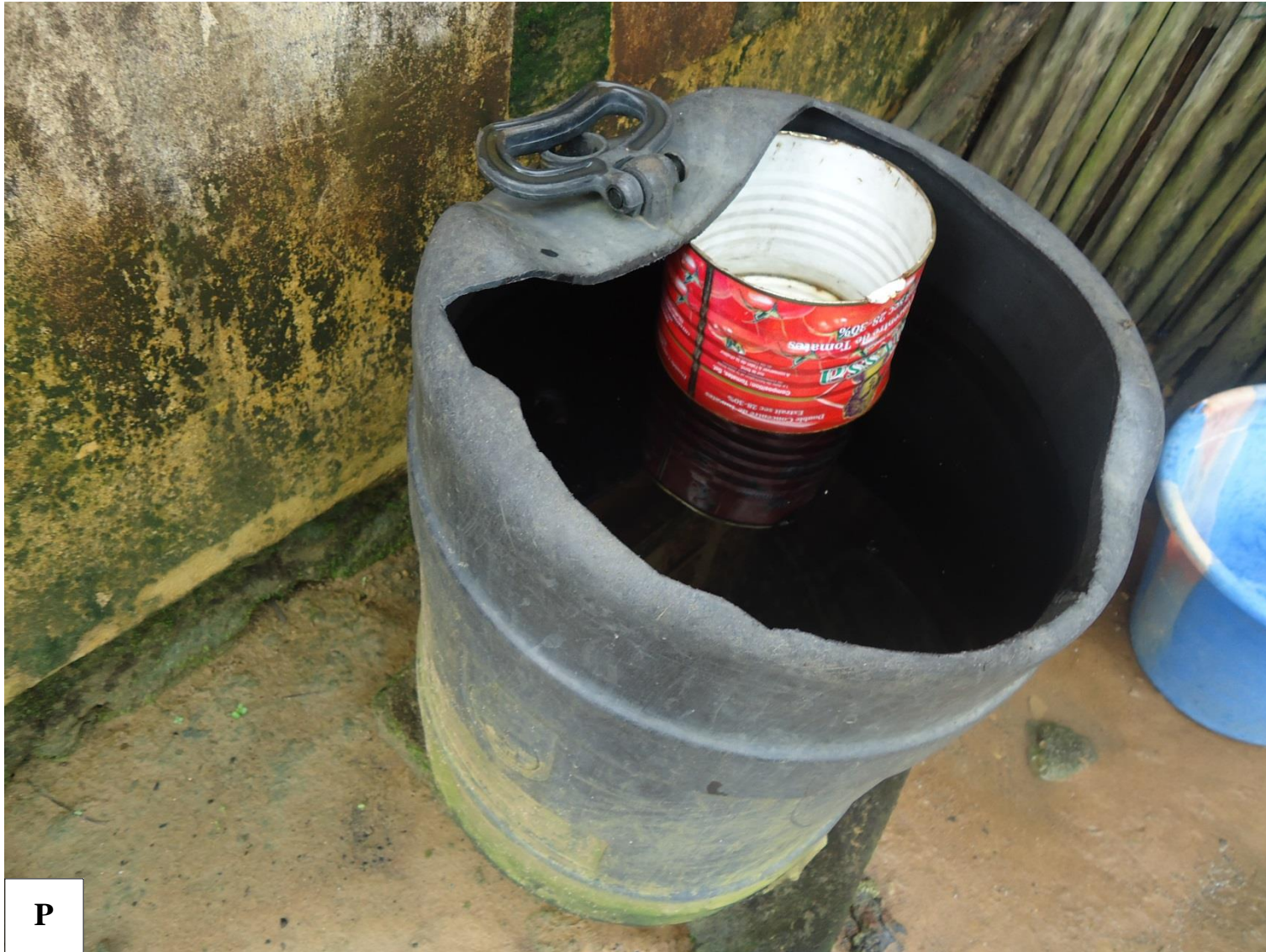

P

Supplement: S1 Fig — The container type often reflects the name of the container and the categories include containers that provide comparable larval habitats as follows: A: rock hole, B: animal detritus, C: leaf, D: fruit husks, E: bamboo, F: tree hole, G: clay pot, H: wood-container, I: metallic pot, J: traps, K: discarded container, L: tire, M: vehicle tank, N: vehicle carcasses, O: building tool, P: water storage container. (PDF) [file pntd.0005751.s001.pdf]

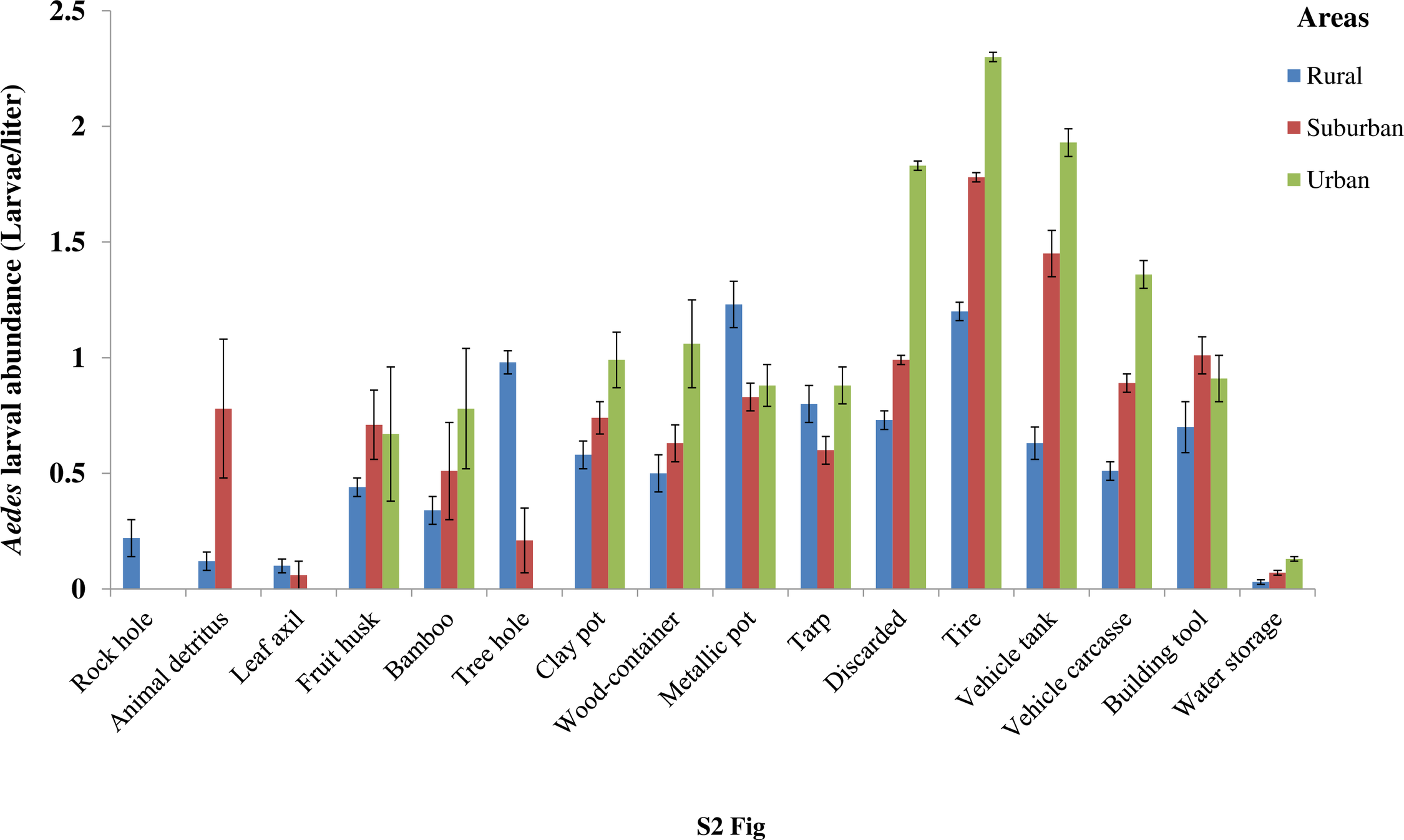

Supplement: S2 Fig — Error bars show the standard error (SE). (TIF) [file pntd.0005751.s002.tif]

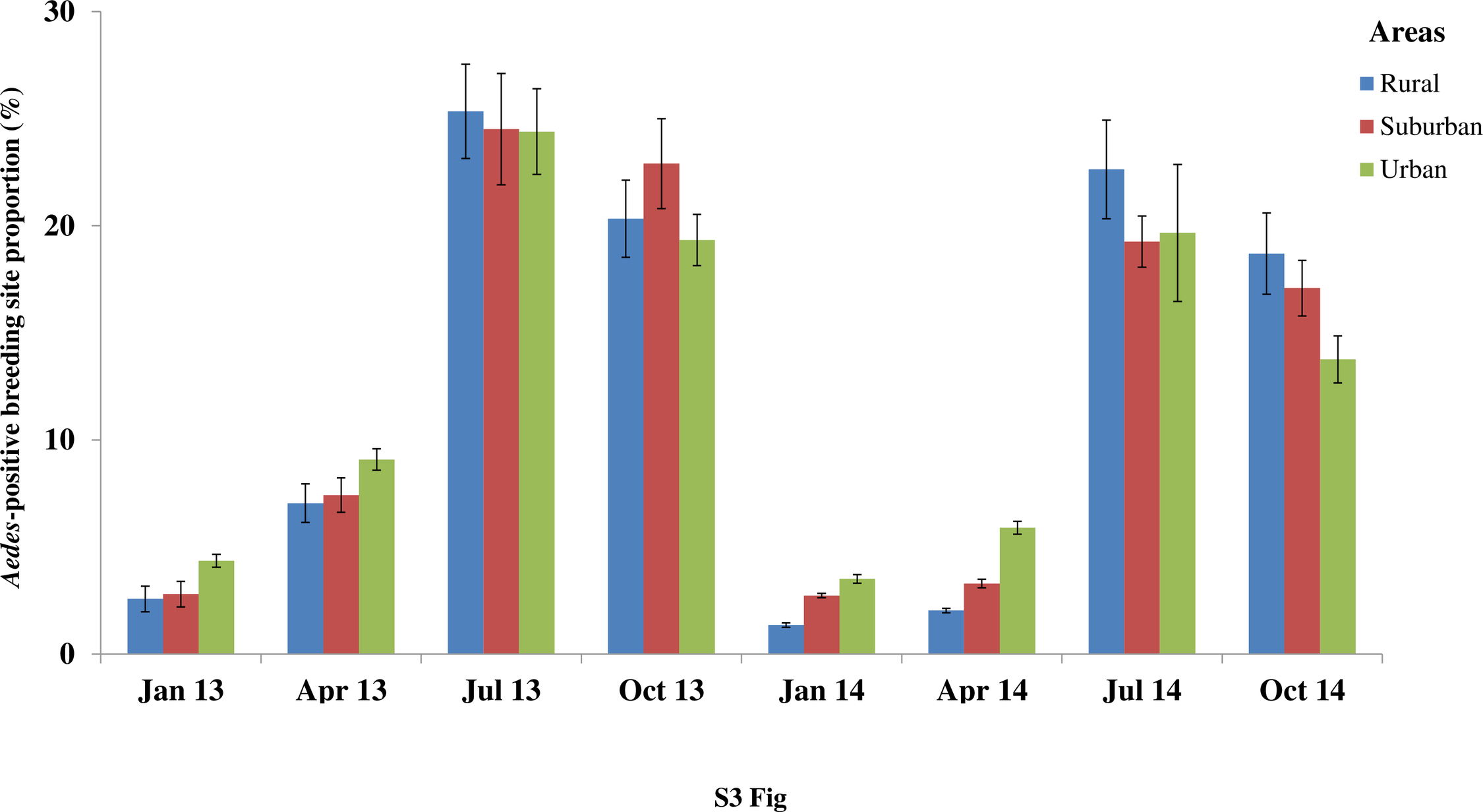

Supplement: S3 Fig — Error bars show the standard error (SE). (TIF) [file pntd.0005751.s003.tif]
