# Supplementary material for: Urbanization is a main driver for the larval ecology of Aedes mosquitoes in arbovirus-endemic settings in south-eastern Côte d'Ivoire
Source: PLoS Negl Trop Dis. 2017 Jul 13;11(7):e0005751. doi: 10.1371/journal.pntd.0005751 (PMC5526600; doi:10.1371/journal.pntd.0005751)
Supplement: S1 Table — (DOCX) [file pntd.0005751.s004.docx]

| **S1 Table. Dynamics of *Aedes* mosquito breeding sites in the rural, suburban and urban areas in south-eastern Côte d’Ivoire from January 2013 to October 2014** | | | | | | | | | | | | |
| --- | --- | --- | --- | --- | --- | --- | --- | --- | --- | --- | --- | --- |
| **Breeding site** | **Rural** | | | | **Suburban** | | | | **Urban** | | | |
|  | **N** | **n** | **FP (%)** | **PP (%)** | **N** | **n** | **FP (%)** | **PP (%)** | **N** | **n** | **FP (%)** | **PP (%)** |
| **Natural** |  |  |  |  |  |  |  |  |  |  |  |  |
| Rock hole | 42 | 6 | 14.3 | 0.8 | 0 | 0 | na | 0.0 | 0 | 0 | na | 0.0 |
| Animal detritus | 82 | 8 | 9.8 | 1.1 | 6 | 3 | 50.0 | 0.2 | 2 | 0 | 0.0 | 0.0 |
| Leaf axil | 151 | 11 | 7.3 | 1.5 | 19 | 3 | 15.8 | 0.2 | 6 | 0 | 0.0 | 0.0 |
| Fruit husk | 195 | 59 | 30.3 | 8.0 | 26 | 11 | 42.3 | 0.8 | 8 | 3 | 37.5 | 0.1 |
| Bamboo | 45 | 17 | 37.8 | 2.3 | 15 | 4 | 26.7 | 0.3 | 9 | 4 | 44.4 | 0.2 |
| Tree hole | 69 | 62 | 89.9 | 8.4 | 11 | 2 | 18.2 | 0.1 | 0 | 0 | na | 0.0 |
| **Total** | **584** | **163** | **27.9** | **22.1** | **77** | **23** | **29.9** | **1.6** | **25** | **7** | **28.0** | **0.3** |
| **Traditional** |  |  |  |  |  |  |  |  |  |  |  |  |
| Clay pot | 101 | 44 | 43.6 | 6.0 | 90 | 43 | 47.8 | 3.0 | 29 | 18 | 62.1 | 0.8 |
| Wood | 69 | 24 | 34.8 | 3.3 | 67 | 31 | 46.3 | 2.2 | 14 | 8 | 57.1 | 0.4 |
| Metallic pot | 44 | 27 | 61.4 | 3.7 | 105 | 61 | 58.1 | 4.3 | 48 | 37 | 77.1 | 1.7 |
| **Total** | **214** | **95** | **44.4** | **12.9** | **262** | **135** | **51.5** | **9.5** | **91** | **63** | **69.2** | **2.9** |
| **Industrial** |  |  |  |  |  |  |  |  |  |  |  |  |
| Tarp | 66 | 41 | 62.1 | 5.6 | 132 | 53 | 40.2 | 3.7 | 95 | 46 | 48.4 | 2.2 |
| Discarded | 254 | 104 | 40.9 | 14.1 | 745 | 417 | 56.0 | 29.2 | 767 | 601 | 78.4 | 28.1 |
| Tire | 324 | 183 | 56.5 | 24.8 | 696 | 525 | 75.4 | 36.8 | 1236 | 1087 | 87.9 | 50.9 |
| Vehicle tank | 84 | 41 | 48.8 | 5.6 | 46 | 34 | 73.9 | 2.4 | 94 | 77 | 81.9 | 3.6 |
| Carcasses | 171 | 68 | 39.8 | 9.2 | 237 | 133 | 56.1 | 9.3 | 131 | 91 | 69.5 | 4.3 |
| Building tool | 38 | 16 | 42.1 | 2.2 | 58 | 34 | 58.6 | 2.4 | 39 | 23 | 59.0 | 1.1 |
| Water storage | 688 | 27 | 3.9 | 3.7 | 816 | 74 | 9.1 | 5.2 | 896 | 141 | 15.7 | 6.6 |
| **Total** | **1625** | **480** | **29.5** | **65.0** | **2730** | **1270** | **46.5** | **88.9** | **3258** | **2066** | **63.4** | **96.7** |
| **Artificial** | **1839** | **575** | **31.3** | **77.9** | **2992** | **1405** | **47.0** | **98.4** | **3349** | **2129** | **63.6** | **99.7** |
| **TOTAL** | **2423** | **738** | **30.5** | **100** | **3069** | **1428** | **46.5** | **100** | **3374** | **2136** | **63.3** | **100** |
| N: number of wet containers, n: number of *Aedes*-positive breeding sites, FP: frequency of positive breeding sites among wet containers, PP: proportion of each *Aedes* breeding site type among *Aedes*-positive containers. | | | | | | | | | | | | |
