# Supplementary material for: Urbanization is a main driver for the larval ecology of Aedes mosquitoes in arbovirus-endemic settings in south-eastern Côte d'Ivoire
Source: PLoS Negl Trop Dis. 2017 Jul 13;11(7):e0005751. doi: 10.1371/journal.pntd.0005751 (PMC5526600; doi:10.1371/journal.pntd.0005751)
Supplement: S2 Table — (DOCX) [file pntd.0005751.s005.docx]

| **S2 Table. Seasonal variations in *Aedes* mosquito breeding site positivity in the rural, suburban and urban areas in south-eastern Côte d’Ivoire from January 2013 to October 2014** | | | | | | | | | | | | | | | | | | | | | | | | |
| --- | --- | --- | --- | --- | --- | --- | --- | --- | --- | --- | --- | --- | --- | --- | --- | --- | --- | --- | --- | --- | --- | --- | --- | --- |
| **Breeding site** | **Rural** | | | | | | | | **Suburban** | | | | | | | | **Urban** | | | | | | | |
|  | **Dry season** | | | | **Rainy season** | | | | **Dry season** | | | | **Rainy season** | | | | **Dry season** | | | | **Rainy season** | | | |
|  | **N** | **n** | **FP** | **PP** | **N** | **n** | **FP** | **PP** | **N** | **n** | **FP** | **PP** | **N** | **n** | **FP** | **PP** | **N** | **n** | **FP** | **PP** | **N** | **n** | **FP** | **PP** |
| **Natural** |  |  |  |  |  |  |  |  |  |  |  |  |  |  |  |  |  |  |  |  |  |  |  |  |
| Rock hole | 6 | 1 | 16.7 | 1.0 | 36 | 5 | 13.9 | 0.8 | 0 | 0 | na | 0.0 | 0 | 0 | na | 0.0 | 0 | 0 | na | 0 | 0 | 0 | na | 0.0 |
| Animal detritus | 2 | 0 | 0.0 | 0.0 | 80 | 8 | 10.0 | 1.2 | 2 | 0 | 0.0 | 0.0 | 4 | 3 | 75.0 | 0.3 | 0 | 0 | na | 0 | 2 | 0 | 0.0 | 0.0 |
| Leaf axil | 13 | 0 | 0.0 | 0.0 | 138 | 11 | 8.0 | 1.7 | 3 | 0 | 0.0 | 0.0 | 16 | 3 | 18.8 | 0.3 | 2 | 0 | 0 | 0 | 4 | 0 | 0.0 | 0.0 |
| Fruit husk | 12 | 2 | 16.7 | 2.1 | 183 | 57 | 31.1 | 8.9 | 2 | 0 | 0.0 | 0.0 | 24 | 11 | 45.8 | 0.9 | 2 | 0 | 0.0 | 0.0 | 6 | 3 | 50.0 | 0.2 |
| Bamboo | 3 | 1 | 33.3 | 1.0 | 42 | 16 | 38.1 | 2.5 | 2 | 0 | 0.0 | 0.0 | 13 | 4 | 30.8 | 0.3 | 0 | 0 | na | 0.0 | 9 | 4 | 44.4 | 0.2 |
| Tree hole | 11 | 9 | 81.8 | 9.4 | 58 | 53 | 91.4 | 8.3 | 2 | 1 | 50.0 | 0.4 | 9 | 1 | 11.1 | 0.1 | 0 | 0 | na | 0 | 0 | 0 | na | 0.0 |
| **Total** | **47** | **13** | **27.7** | **13.5** | **537** | **150** | **27.9** | **23.4** | **11** | **1** | **9.1** | **0.4** | **66** | **22** | **33.3** | **1.8** | **4** | **0** | **0.0** | **0.0** | **21** | **7** | **33.3** | **0.4** |
| **Traditional** |  |  |  |  |  |  |  |  |  |  |  |  |  |  |  |  |  |  |  |  |  |  |  |  |
| Clay pot | 21 | 11 | 52.4 | 11.5 | 80 | 33 | 41.3 | 5.1 | 15 | 3 | 20.0 | 1.3 | 75 | 40 | 53.3 | 3.3 | 5 | 2 | 40.0 | 0.4 | 24 | 16 | 66.7 | 1.0 |
| Wood | 6 | 2 | 33.3 | 2.1 | 63 | 22 | 34.9 | 3.4 | 7 | 1 | 14.3 | 0.4 | 60 | 30 | 50.0 | 2.5 | 2 | 0 | 0.0 | 0.0 | 12 | 8 | 66.7 | 0.5 |
| Metallic pot | 3 | 1 | 33.3 | 1.0 | 41 | 26 | 63.4 | 4.0 | 17 | 8 | 47.1 | 3.4 | 88 | 53 | 60.2 | 4.4 | 9 | 4 | 44.4 | 0.8 | 39 | 33 | 84.6 | 2.0 |
| **Total** | **30** | **14** | **46.7** | **14.6** | **184** | **81** | **44.0** | **12.6** | **39** | **12** | **30.8** | **5.2** | **223** | **123** | **55.2** | **10.3** | **16** | **6** | **37.5** | **1.2** | **75** | **57** | **76.0** | **3.5** |
| **Industrial** |  |  |  |  |  |  |  |  |  |  |  |  |  |  |  |  |  |  |  |  |  |  |  |  |
| Tarp | 12 | 3 | 25.0 | 3.1 | 54 | 38 | 70.4 | 5.9 | 5 | 3 | 60.0 | 1.3 | 127 | 50 | 39.4 | 4.2 | 23 | 4 | 17.4 | 0.8 | 72 | 42 | 58.3 | 2.5 |
| Discarded | 45 | 10 | 22.2 | 10.4 | 209 | 94 | 45.0 | 14.6 | 132 | 59 | 44.7 | 25.4 | 613 | 358 | 58.4 | 29.9 | 178 | 120 | 67.4 | 24.7 | 589 | 481 | 81.7 | 29.2 |
| Tire | 66 | 33 | 50.0 | 34.4 | 258 | 150 | 58.1 | 23.4 | 163 | 102 | 62.6 | 44.0 | 533 | 423 | 79.4 | 35.4 | 299 | 240 | 80.3 | 49.4 | 937 | 847 | 90.4 | 51.3 |
| Vehicle tank | 4 | 2 | 50.0 | 2.1 | 80 | 39 | 48.8 | 6.1 | 3 | 2 | 66.7 | 0.9 | 43 | 32 | 74.4 | 2.7 | 13 | 6 | 46.2 | 1.2 | 81 | 71 | 87.7 | 4.3 |
| Carcasses | 39 | 3 | 7.7 | 3.1 | 132 | 65 | 49.2 | 10.1 | 52 | 16 | 30.8 | 6.9 | 185 | 117 | 63.2 | 9.8 | 35 | 18 | 51.4 | 3.7 | 96 | 73 | 76.0 | 4.4 |
| Building tool | 5 | 2 | 40.0 | 2.1 | 33 | 14 | 42.4 | 2.2 | 15 | 6 | 40.0 | 2.6 | 43 | 28 | 65.1 | 2.3 | 28 | 19 | 67.9 | 3.9 | 11 | 4 | 36.4 | 0.2 |
| Water storage | 318 | 16 | 5.0 | 16.7 | 370 | 11 | 3.0 | 1.7 | 386 | 31 | 8.0 | 13.4 | 430 | 43 | 10.0 | 3.6 | 409 | 73 | 17.8 | 15.0 | 487 | 68 | 14.0 | 4.1 |
| **Total** | **489** | **69** | **14.1** | **71.9** | **1136** | **411** | **36.2** | **64.0** | **756** | **219** | **29.0** | **94.4** | **1974** | **1051** | **53.2** | **87.9** | **985** | **480** | **48.7** | **98.8** | **2273** | **1586** | **69.8** | **96.1** |
| **Artificial** | **519** | **83** | **16.0** | **86.5** | **1320** | **492** | **37.3** | **76.6** | **795** | **231** | **29.1** | **99.6** | **2197** | **1174** | **53.4** | **98.2** | **1001** | **486** | **48.6** | **100** | **2348** | **1643** | **70.0** | **99.6** |
| **TOTAL** | **566** | **96** | **17.0** | **100** | **1857** | **642** | **34.6** | **100** | **806** | **232** | **28.8** | **100** | **2263** | **1196** | **52.9** | **100** | **1005** | **486** | **48.4** | **100** | **2369** | **1650** | **69.6** | **100** |
| N: number of wet containers, n: number of *Aedes*-positive breeding sites, FP: frequency of positive breeding sites among wet containers, PP: proportion of each *Aedes* breeding site type among *Aedes*-positive containers. The units of FP and PP are percentage (%). | | | | | | | | | | | | | | | | | | | | | | | | |
